# Alkali salts of amino acids as alkaline additives for neutralization of acidic corrosion inhibitors

**DOI:** 10.1007/s00726-023-03260-x

**Published:** 2023-03-10

**Authors:** Tim Naundorf, Tom Seddig, Erik Ruf, Laurens Ballentin, Helmut Kipphardt, Wolfgang Maison

**Affiliations:** 1 Department of Chemistry, Universität Hamburg, Bundesstraße 45, 20146 Hamburg, Germany; 2 Metall-Chemie Technologies GmbH, Herrengraben 30, 20459 Hamburg, Germany

**Keywords:** Amino acids, Corrosion inhibitors, Co-leaching, Scale inhibitors, Metalworking fluids, Electrochemistry

## Abstract

We present alkali salts of amino acids as functional, non-toxic, non-hazardous, non-volatile, chemically stable, and cheap alkaline additives for common acidic corrosion inhibitors. The resulting mixtures have been evaluated for Co, Ni, and Cu leaching and were analyzed by chip filter assay, potentiodynamic polarization measurements, electrochemical impedance measurements, and gravimetry for corrosion protection of iron and steel in aqueous environment at slightly alkaline pH. Leaching of Co and Ni was found to depend on the corresponding complex stabilities. Taurine (Tau) as well as aminohexanoic acid (AHX) leads to low leaching of Co and Ni. Particularly AHX is an attractive low leaching additive leading to lower Co and Ni concentrations in solution than currently used amino alcohols. Glu and Tau were found to synergistically interact with several acidic corrosion inhibitors of the carboxylic acid and the phosphonic acid type. Tau had a particularly positive impact on the protective properties of carboxyphosphonic acids. Glu had also a positive effect on the anti-corrosive properties of several acidic corrosion inhibitors and served as an anti-scalant. Alkali salts of Glu and Tau might thus serve as commercially and ecologically attractive substitutes for current alkaline additives to acidic corrosion inhibitors.

**Supplementary Information:**

The online version contains supplementary material available at 10.1007/s00726-023-03260-x.

## Introduction

**Fig. 1:**
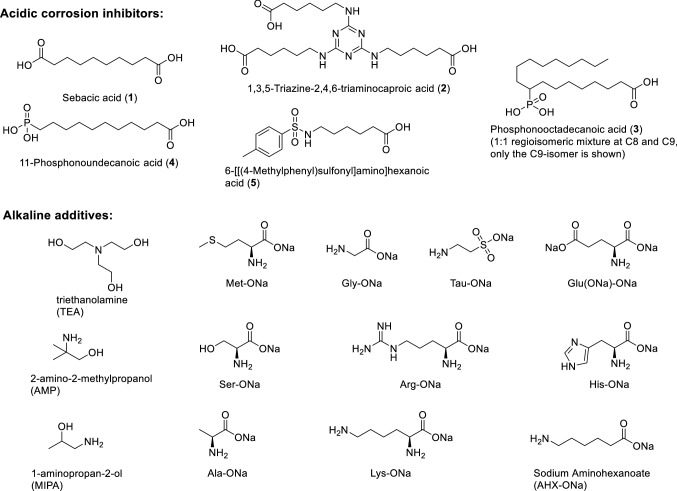
Chemical structures of acidic CIs and alkaline additives used in this work

2.4 billion liters of metalworking fluids (MWFs) were utilized worldwide in 2017 (Najiha et al. 2016). It is estimated that MWFs cause up to 16.9% of the production costs in the metal-processing industry (Lotierzo et al. 2016; Najiha et al. 2016). They play a significant role in processes like drilling, forming, grinding, or cutting of metals in reducing friction between tool and workpiece and as coolants. MWFs are therefore crucial to avoid thermal damage of work pieces and reduce wear on the tool (Brinksmeier et al. 2015). On the other hand, many of their ingredients have had serious impact on the environment and the health of metal workers (Bay et al. 2010). Many efforts were therefore undertaken to develop safer and more environmentally friendly MWFs (Rudnick 2005; Umoren et al. 2019). Following the principles of green chemistry (Anastas and Kirchhoff 2002; Kümmerer 2007), some of the key aspects in this context are: elimination of hazardous chemicals, simplification of MWF composition, and increased resource efficiency (currently, most ingredients are derived from mineral oil), including longer service life of tools and MWFs, reuse, and recovery of MWFs (Bay et al. 2010; Brinksmeier et al. 2015). They can be divided in three groups according to their formulation: oil-based, water-based, and emulsions (Byers and Byers 2017). Due to its high coefficient of heat transfer, aqueous MWFs are particularly attractive if good cooling properties are needed. However, the use of aqueous MWFs demands addition of corrosion inhibitors (CIs) to protect workpiece and tool. Next to several other additives, such as extreme pressure additives, anti-wear additives, antifoam additives, biocides, and emulsifiers, CIs are thus major components of MWFs and account typically for about 1–5 wt%. Many CIs are carboxylic acids (Kuznetsov 2016) or phosphonic acids (Abohalkuma et al. 2016; Byers and Byers 2017; Spikes 2004). Because the pH of MWFs is typically slightly alkaline (pH = 8.0–9.5) to reduce corrosion and microbial load, acidic CIs are neutralized with alkaline additives such as amines or amino alcohols of low molecular weight, which are often used in excess to buffer the pH in the desired slightly alkaline region (Sanyal 1981). Typical examples include 2-amino-2-methylpropanol (AMP), 1-aminopropan-2-ol (MIPA), and tri-ethanol-amine (TEA) (Fig. 1), which are all water-soluble primary or tertiary amines (Bennett and Bennett 1984; Byers and Byers 2017; Lotierzo et al. 2016). Secondary amines tend to react with nitrogen oxides and nitrite to carcinogenic *N*-nitrosamines and are therefore not used in this context. In addition to their buffering capacity at favorable slightly alkaline pH values, vicinal amino alcohols have anti-corrosive properties, which have been assigned to the formation of protective layers (Jamil et al. 2003; Welle et al. 1997). Most studies on anti-corrosive properties of amines (and amino alcohols) have been performed in acidic media and are therefore hardly comparable to the situation in alkaline MWFs. However, some studies at alkaline pH values (mostly related to corrosion in concrete (Soylev and Richardson 2008)) have been performed, and weak anti-corrosive properties for amino alcohols were reported, only when high concentrations of these compounds were applied (Ormellese et al. 2009; Vyrides et al. 2013). Besides, synergistic effects of amino alcohols on the anti-corrosive efficacy of acidic CIs in MWFs have been reported in some cases (Kern and Landolt 2001; Ochoa et al. 2004). In other cases, no synergistic effects were observed (Brenna et al. 2017). The chemical mechanism behind the corrosion protective synergistic effects of amino alcohols is not completely understood. It has been postulated, that alkaline additives act by creating active sites on the metal surface, by a fast interaction with hydrated iron oxide films and create thus favorable anchoring points for the stable immobilization of the acidic CIs through chemisorption (Felhősi et al. 2002; Kern and Landolt 2001; Ochoa et al. 2005). Additional reasons might include their influence on surface activity of the neutralized CIs by formation of salts and/or their chelating ability for dissolved metal cations.

Despite their common use, many amino alcohols have major drawbacks. TEA, for example, has been linked to allergic contact dermatitis (Bruze et al. 1995; Chu and Sun 2001), seawater ecotoxicity (Libralato et al. 2010), and carcinogenic activity (Fiume et al. 2013). In addition, it is listed on the EU “Control List of Dual-Use Items in Annex I to Regulation (EC) No 428/2009” which entails further regulation and higher cost. In addition, some amino alcohols like AMP or MIPA are relatively volatile. Substitution of these compounds by less toxic and environmentally friendly alkaline additives would therefore be desirable. In this context, amino acids combine a number of favorable features. They are non-toxic (many are edible), non-hazardous, non-volatile, chemically stable, and available at low cost in large tonnages often from bio-renewables (Beerthuis et al. 2015). Amino acids have therefore been frequently used in the context of green chemistry (Shirini and Daneshvar 2016; Somorjai et al. 2009; Yang et al. 2016) and investigated as green acidic CIs (Verma et al. 2021, 2018). However, the anti-corrosive properties of amino acids at alkaline pH values are limited (Kasprzhitskii et al. 2021; León González 2021; Ormellese et al. 2009) and only selected amino phosphonic acids are used commercially as antiscalants and CIs mostly in combination with zinc salts (Moschona et al. 2018). Anticorrosive properties of amino carboxylic acids in acidic media have also been found to be improved by addition of Lewis acids such as Zn^2+^ or surfactants (Mobin et al. 2017; Pech-Canul and Chi-Canul 1999; Zhang et al. 2016). To the best of our knowledge, no attempts to use amino acids as alkaline additives for organic acidic CIs have been reported yet.

**Scheme 1:**
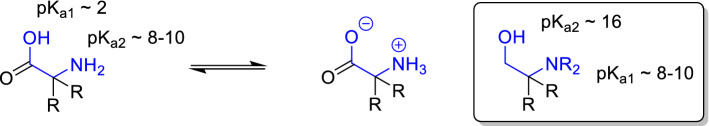
Structural comparison and typical pka values for α-amino carboxylic acids and vicinal amino alcohols

A closer look at the structures of amino (carboxylic) acids and amino alcohols in Scheme 1 reveals structural similarities and striking differences in acidities, which apply (although to different degrees) not only to amino carboxylic acids depicted but also to related derivatives such as amino sulfonic acids. As indicated by pK_a_ values, the most important difference of amino alcohols and amino acids is the higher acidity of the acid group compared to the alcohol group. Amino acids are therefore deprotonated at neutral pH, and the resulting carboxylate salts will thus increase the conductivity of solutions compared to non-ionic alkaline additives. Similar to many vicinal amino alcohols, α-amino carboxylates are good chelate ligands for alloying metals, forming water-soluble metal complexes. They might thus be corrosive to certain metals and contribute to leaching of these metals into the solution. Although undesired in possible applications of amino acids in MWFs, the latter aspect has been used for cobalt leaching in battery recycling with glycinate (Nayaka et al. 2016).

In this study, we describe the application of amino acid sodium salts as alkaline additives for selected CIs of the phosphonic acid and carboxylic acid type. Anticorrosive properties of the resulting mixtures were evaluated with a visual chip filter assay, electrochemical measurements, and gravimetry in slightly alkaline media. In addition, we analyzed leaching properties of pure alkaline additives and their mixtures with acidic CIs for Co, Ni, and Cu as important alloying metals.

## Materials and methods

### Chemicals

Chemicals were purchased from Sigma-Aldrich in reagent grade and used as received. Amino acids were converted to their sodium salts through dissolution in water and addition of the appropriate equivalents NaOH, prior to use. Carboxyphosphonic acids **3** and **4** (Fig. 1) were synthesized according to a literature protocol (Ruf et al. 2022).

### Chip filter test

Evaluation of the anti-corrosive properties of the substances for iron was carried out according to DIN 51360–02-A using the aqueous, solution of the acidic Cis, and alkaline additive in either hard water (20dGH: CaCl_2_ 340 mg/L, MgSO_4_ 60.0 mg/L) or 0.5% aqueous NaCl (pH 8.3 ± 0.5) at the appropriate concentration (Tables S2 + S3, see supporting information). 1.2 or 1.5 molar equivalents of the alkaline additive were added for each acidic proton in acidic CIs. As an example, carboxyphosphonic acid **3** contains 3 acidic protons, thus 3.6 or 4.5 molar eq of the alkaline additive were added. 2 mL of the appropriate test solution was incubated with 2.0 g of sieved gray cast iron turnings from Riegger Industriehandel GMBH for 2 h, before rinsing and visual scoring according to DIN 51360–02-A. For data, see supporting information: Tables S2 + S3.

### Evaluation of metal leaching

225 mg of the appropriate metal–powder (Co: powder, < 150 μm, ≥ 99.9% trace metals basis from Sigma-Aldrich; Cu: powder, < 425 μm, 99.5% trace metals basis from Sigma-Aldrich; Ni: powder 100 mesh/ 99.9% from Chempur) was suspended in 15 mL of an aqueous solution of the appropriate alkaline additive (1wt%, amino acids were used as sodium salts) or a mixture of an acidic CI and sodium-6-aminohexanoate (Na-AHX). The mixture was heated to reflux for 24 h. 1.5 eq Na-AHX were added for each acidic proton in acidic CIs. As an example, carboxyphosphonic acid **3** contains 3 acidic protons, thus 4.5 molar eq Na-AHX were added as alkaline additive. After heating, the suspension was cooled to room temperature, filtered, and the metal concentration in solution measured directly via flame atomic absorption spectroscopy (FAAS). Reported metal concentrations are mean values of triplicate measurements.

### Electrochemistry

**Fig. 2:**
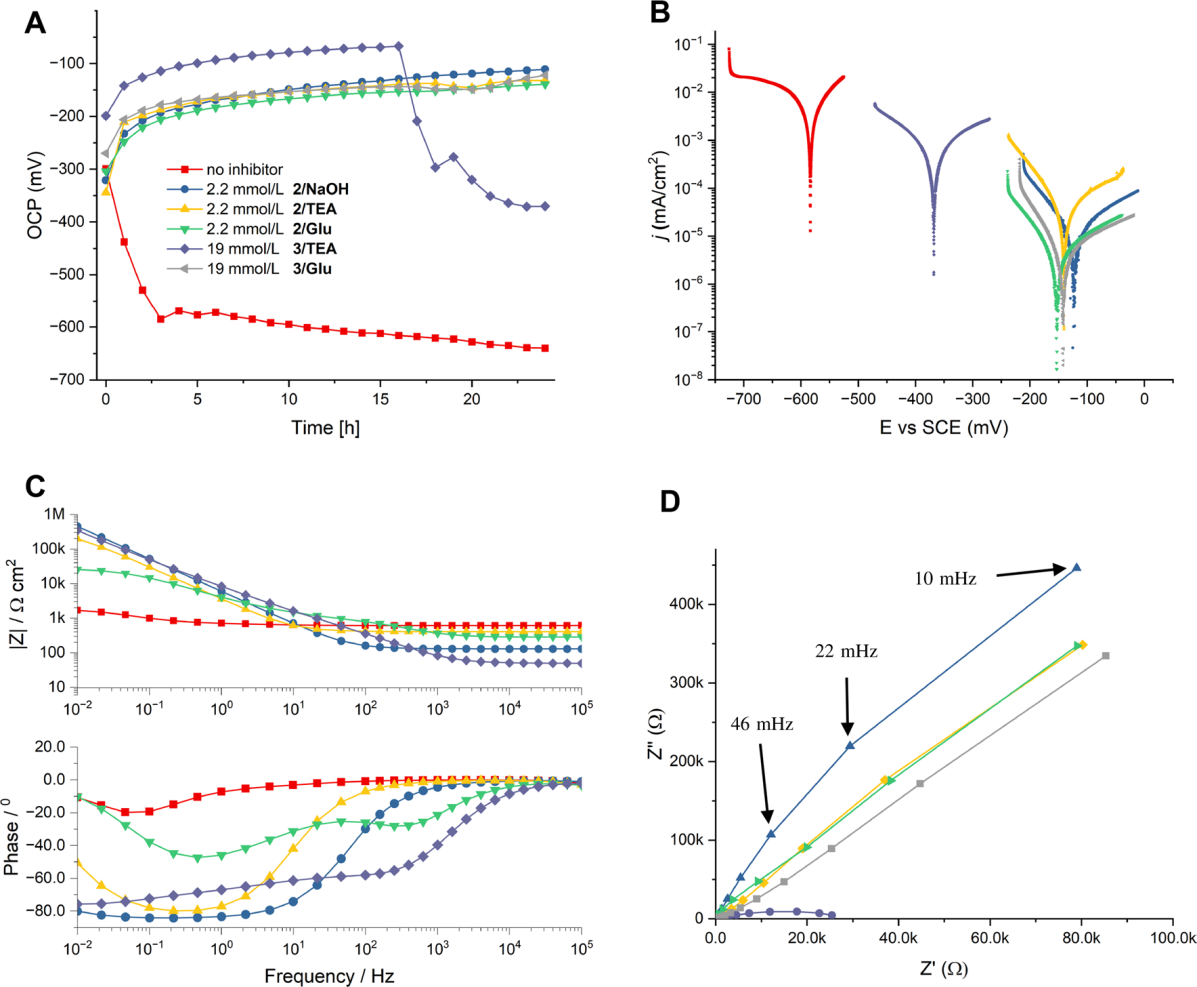
Electrochemical measurements of steel (SJR235) in hard water (20dGH, pH 8.3 ± 0.5) with tricarboxylic acid **2** or carboxyphosphonic acid **3** and NaOH, TEA and Glu (disodium glutamate) as alkaline additives. **A** Development of open circuit potential (OCP) with immersion time; **B** potentiodynamic polarization curves after 24 h immersion; **C** Bode plots of electrochemical impedance measurements. **D** Nyquist plots of electrochemical impedance measurements

**Fig. 3:**
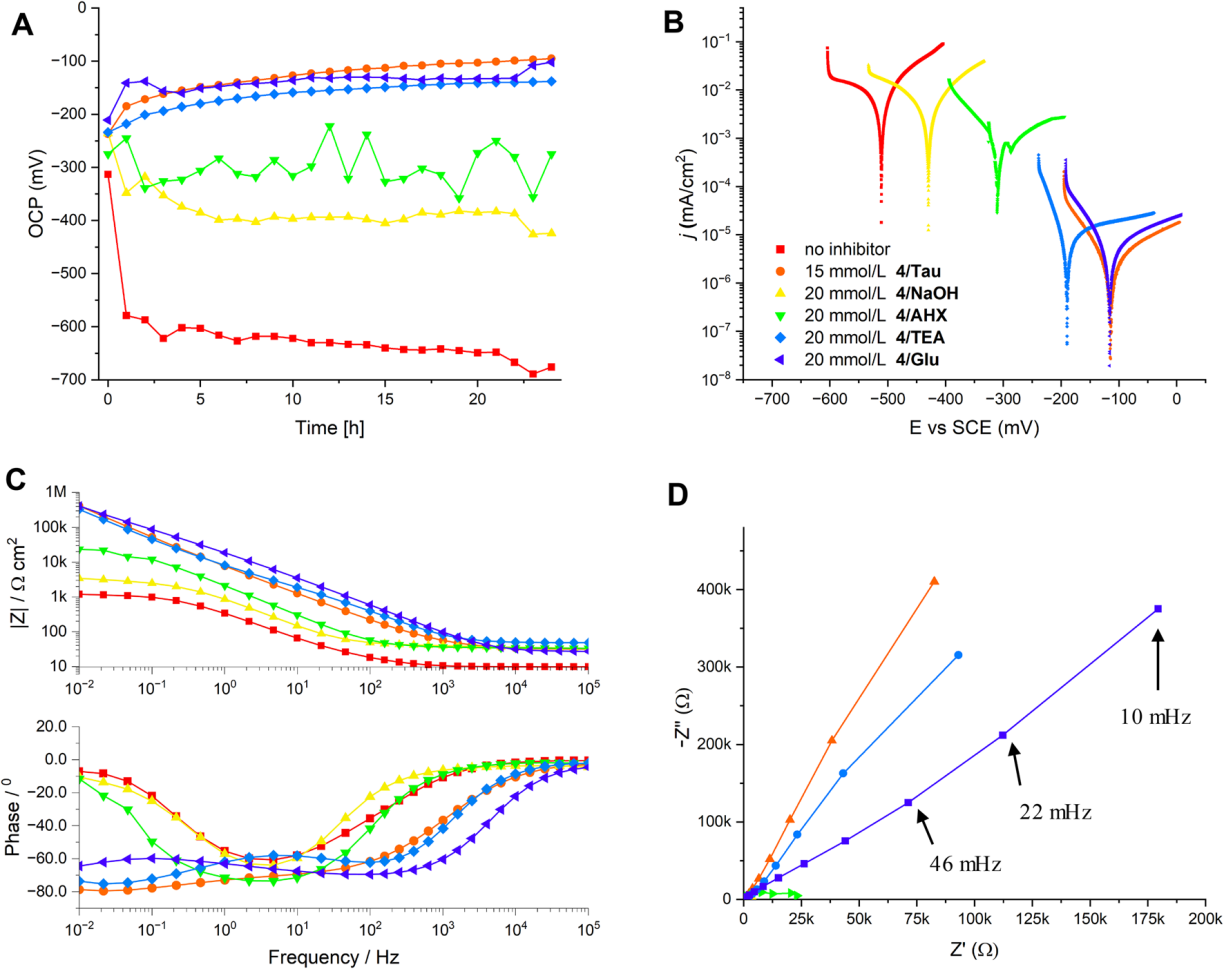
Electrochemical measurements of steel (SJR235) in aqueous NaCl (0.5wt.%, pH 8.3 ± 0.5) with carboxyphosphonic acid **4** and NaOH, TEA, Tau (sodium salt), AHX (sodium salt) and Glu (disodium salt) as alkaline additives. **A** Development of open circuit potential (OCP) with immersion time; **B** potentiodynamic polarization curves after 24 h immersion; **C** Bode plots of electrochemical impedance measurements; **D** Nyquist plots of electrochemical impedance measurements

**Fig. 4:**
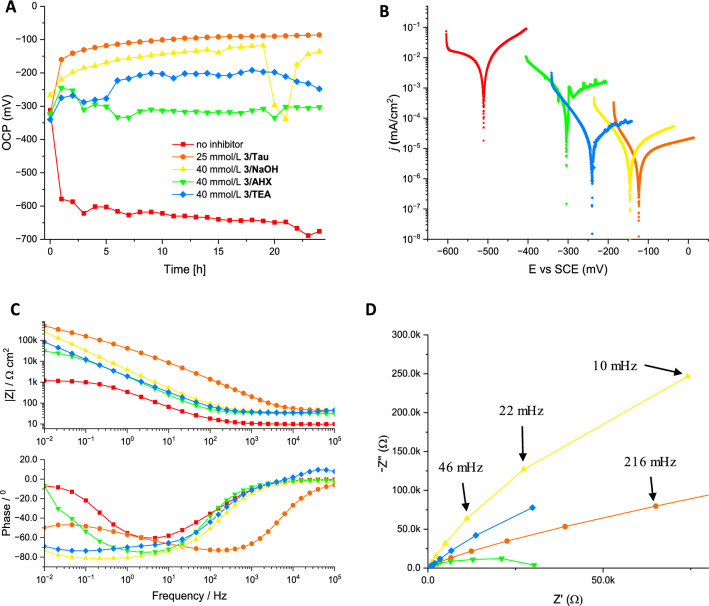
Electrochemical measurements of steel (SJR235) in aqueous NaCl (0.5wt.%, pH 8.3 ± 0.5) with carboxyphosphonic acid **3** and NaOH, TEA, Tau (sodium salt), AHX (sodium salt) and Glu (disodium salt) as alkaline additives. **A** Development of open circuit potential (OCP) with immersion time; **B** potentiodynamic polarization curves after 24 h immersion; **C** Bode plots of electrochemical impedance measurements. **D** Nyquist plots of electrochemical impedance measurements

Quadratic S235JR steel slides (30 × 30 × 2 mm, obtained from Franz Krüppel GmbH & Co KG, ingredients according to DIN EN 10,025–2: C = 0.17%, Mn = 1.40%, P = 0.035%, S = 0.035%, N = 0.012%, Cu = 0.55%) for the electrochemical assays were treated according to ASTM G1 C3.5 and cleaned in an ultrasonic bath (HCl 6 m; Urotropine 7 g/L) for 10 min and then dried in a nitrogen stream. Potentiodynamic polarization curves were measured with a Gamry ParaCell setup. The samples were used as a working electrode and were exposed to the electrolyte with an O-ring sealed opening with a diameter of 1 cm^2^. The counter electrode was graphite and the reference electrode was Ag/AgCl in a 3 M KCl solution (SCE) which has a shift of + 210 mV compared with a standard hydrogen electrode (SHE). Open circuit measurements were taken over 24 h. Within this time span, the open circuit potential was measured every hour for 200 s. Each data point in Figs. 2a, 3, 4a is a median value of these 200 s. Impedance measurements were carried out using AC signals of a 10 mV potential perturbation and the scanning frequency range was 100 kHz to 0.01 Hz with 5 point/decade. The polarization scan was between − 100 mV and + 100 mV against the open circuit potential with a scan rate of 0.1 mV/s. The evaluation for the potentiodynamic measurements was performed according to a single determination in the specified measuring range. The polarization resistance (*R*_*p*_) was determined using the slope at 0 A between − 20 mV and + 20 mV of the corrosion potential. The electrolyte was either hard water (20dGH: CaCl_2_ 340 mg/L, MgSO_4_ 60.0 mg/L, conductivity at 25 °C: 861 μS/cm) or NaCl solution containing the CI and the appropriate alkaline additive (final pH 8.3 ± 0.5). The polarization measurement was performed after 24 h exposure at room temperature. Data were processed with the program Gamry Echem Analyst (Version 7.8.2). The percentage inhibition efficiency %IE_corr_ was calculated as follows:

1 $${\mathrm{IE}}_{\mathrm{corr}}=\frac{{I}_{\mathrm{corr},\mathrm{ uninhibited}}-{I}_{\mathrm{corr},\mathrm{ inhibited}}}{{I}_{\mathrm{corr},\mathrm{ uninhibited}}}100\%$$

with *I*_corr_ values from the uninhibited (hard water 20dGH, pH 7.9–8.8) and inhibited solutions, respectively. Corrosion rates were calculated according to literature (Groysman 2010).

### Gravimetric corrosion test

Rectangular S235JR steel coupons (30 × 10 × 3 mm, obtained from Rocholl GMBH, ingredients according to DIN EN 10025–2: C = 0.17%, Mn = 1.40%, P = 0.035%, S = 0.035%, N = 0.012%, Cu = 0.55%) were treated according to ASTM G1-03(2017)e1 and cleaned in an ultrasonic bath (HCl 6 M; Urotropine 7 g/L) for 10 min and then dried in a nitrogen stream. Afterward, the steel coupons were immersed in test solutions for 12 weeks at room temperature with regular substitution of evaporated water by the addition of demineralized water. Test solutions were obtained by dissolving the appropriate acidic CI in 2% aqueous NaCl and addition of 1.5 eq alkaline additive for each acidic proton in acidic CIs. As an example, carboxyphosphonic acid **3** contains 3 acidic protons, thus 4.5 eq alkaline additives were added. The pH of the resulting test solutions was 8.3 ± 0.5. The corrosion rate *C*_*R*_ in µm/y was calculated as follows:

2 $${C}_{R}=\frac{\Delta m}{A \cdot d \cdot t}365 \cdot {10}^{9}$$

with Δ*m* is the weight loss in g, *A* = 840 mm^2^ (sample surface), *d* = 7940 kg/m^3^ (density of steel) and *t* = 84 d (immersion time). Each experiment was performed in triplicate.

## Results and discussion

Amino acids have a number of desirable properties for the use in formulation of MWFs. If used as alkali salts, they serve as mild bases and can thus be used to buffer the pH of common acidic CIs to values around 8–9 (just as the above-mentioned amino alcohols). Several derivatives are readily available in large tonnages at low cost. In addition, most amino acids are non-toxic, non-hazardous, non-volatile, and chemically stable. Following these criteria, we selected a set of simple amino acids for evaluation as alkaline additives to aqueous MWFs. Within this set of amino acids, glycine (Gly) was chosen as the simplest, alanine (Ala) as an alkyl-substituted, arginine (Arg) and lysine (Lys) as basic, glutamate (Glu) as acidic, serine (Ser) and cysteine (Cys) as donor-substituted, and histidine (His) as heteroaromatic proteinogenic amino acid. In addition, 6-amino hexanoic acid (AHX) as a non-proteinogenic higher homologue and taurine (Tau) as a α-amino sulfonic acid were also included. All amino acids were used in the form of their sodium salts.

### Selection of amino acids via chip filter test

For a fast selection of the most promising combinations of amino acids with CIs, we performed a chip filter test on gray cast iron. The chip filter test according to DIN 51,360–2 is a standard method used in industry for evaluation of anti-corrosive properties of MWFs for iron. Briefly, a defined amount of sieved gray cast iron chips is placed on a round paper filter and submerged in a hard water solution of an acidic CI and an alkaline additive. After incubation for 2 h at room temperature and subsequent washing of the filter, corrosion marks are visually detected and scored between 0 (no corrosion marks) and 4 (strong staining of the filter due to corrosion). This assay is operationally very simple and can be easily used for medium throughput screening. In addition, it is quite sensitive, due the large surface area and the high corrosivity of gray cast iron chips. However, the optical detection of staining is slightly subjective and open to misinterpretation regarding staining that is not caused by corrosion. The chip filter test was therefore used for a preselection of promising mixtures only. These were afterward evaluated in more detail using electrochemical measurements and gravimetry. Tables with all corrosion scores obtained by chip filter assay are available in the supporting information (Tables S2 and S3). As a benchmark system, we used an aqueous solution of 1,3,5-triazine-2,4,6-triaminocaproic acid (**2**, 64 mmol/L corresponding to 3 wt%) with TEA (3.6 or 4.5 molar equivalents, respectively) as an alkaline additive. This mixture is used successfully in several commercial MWFs for applications involving steel tools. It has a relatively low pH value of 7.4–7.8 and shows complete corrosion protection (score = 0) in the chip filter assay. In brief, the following observations were made: 1. Organic additives such as TEA are essential for the performance of acidic CIs. When TEA was substituted by NaOH as alkaline additive to CI **2**, a corrosion score of 4 was obtained although the pH of this mixture (pH = 10.3, suggesting improved alkaline corrosion protection) was significantly higher than that of TEA/**2** (pH = 7.4). 2. Sodium salts of Tau, Glu, and AHX were as effective as TEA (scores = 0 at 3.6 molar equivalents) when used as alkaline additives, while other amino acids were less effective (scores = 1–4). 3. Corrosion scores for all amino acids tested were improved to 0–1 with a slight increase in concentration from 3.6 to 4.5 molar equivalents. 4. Amino acids are also compatible with acidic CIs other than **2**. We have tested three commercial CIs **1**, **2**, and **5** containing 1–3 carbocyclic acid groups as well as two recently described carboxyphosphonic acids **3** and **4** (Ruf et al. 2022) and obtained perfect corrosion scores for combinations with sodium salts of Tau, Glu, and AHX in all cases. 5. All amino acids gave perfectly clear and colorless solutions upon formulation with acidic CIs. However, after storage of 1 month at room temperature, the mixtures containing Met showed a yellow color, indicating oxidative degradation processes. 6. Several mixtures with Gly gave an untypical uniform color reaction on filter paper after incubation with gray cast chips lacking the normal contoured staining pattern of corroded iron chips (see supporting information, Fig. S4). We attributed this finding to the formation of colored water-soluble Gly complexes with alloying metals, which might have been formed as a consequence of Gly-mediated metal leaching from gray cast iron chips. Tau, Glu, and AHX showed the most promising properties of all amino acids tested and were selected for further evaluation by electrochemical measurements.

### Leaching properties of amino acids for Co, Ni and Cu

Leaching of alloying metals is an undesired property of MWFs. Cobalt leaching, for example, causes increased wear of tools and is a serious environmental and health hazard (Barceloux 1999). Given the observations made for Gly in the chip filter assay, we analyzed the leaching ability of several amino acids at slightly alkaline pH values. In addition, we evaluated selected mixtures with acidic CIs. As benchmarks, we compared the leaching abilities of amino acids with TEA, AMP, and MIPA. The latter two are marketed as low leaching additives for Co-containing alloys. In a first set of experiments, the appropriate metal powder was suspended with an aqueous solution of the alkaline additive and heated to reflux for 24 h. Dissolved metal concentrations were measured via FAAS and are shown in Table 1.

**Table 1:** Co, Ni and Cu-concentrations after exposure of the appropriate metal powder to aqueous solutions of additives for 24 h at reflux

	Additive^a^	pH	Co (mg/L)	Cu (mg/L)	Ni (mg/L)
1	NaOH	9.6	0.67	≤ 0.20	≤ 0.20
2	TEA	10.2	34.3	1.57	1.44
3	AMP	12.8	7.03	43.2	0.33
4	MIPA	12.6	0.82	26.4	1.32
5	Gly	9.6	2486	24.8	963
6	Sar	9.6	419	70.6	328
7	*N,N-*Dimethyl-Gly	9.6	207	154	75
8	Lys	10.8	501	109	166
9	Ser	9.6	592	0.33	231
10	Glu	9.4	454	86.0	192
11	Pro	9.6	438	239	318
12	AHX	10.1	≤ 0.20	0.81	≤ 0.20
13	Tau	8.7	34.4	5.76	8.92
14	**1**/AHX^d^	9.8	4.04	4.66	84.3
15	**2**/AHX^b^	9.8	3.76	5.53	53.3
16	**3**/AHX^b^	10.0	0.63	0.82	13.4
17	**4**/AHX^c^	9.9	0.72	0.70	152
18	**5**/AHX^b^	9.9	1.19	1.15	135

Metal concentrations were measured as triplicate from the aqueous extracts via flame atomic absorption spectroscopy (FAAS) and are given as mean values

^a^Performed with 1wt% of the alkaline additive. Amino acids were used as sodium salts

^b^Performed with 3wt.% acidic CI and 4.5 molar equivalents AHX sodium salt

^c^Performed with 3wt.% acidic CI and 1.5 molar equivalents AHX sodium salt

^d^Performed with 3wt.% acidic CI and 3 molar equivalents AHX sodium salt

The results confirm the low leaching capability of AMP and MIPA for Co and Ni (Table 1, entries 3–4). TEA in contrast leads to moderate concentrations of dissolved Co and Ni (Table 1, entry 2), which has been noted by other researchers before and attributed to the formation of water-soluble TEA complexes at least for Co (Zhang et al. 2010). For Cu, the trend is inverted and TEA leads to lower amounts of dissolved Cu than AMP and MIPA. Sodium glycinate, on the other hand, leads to exceptionally high levels of Co and Ni in solution and can thus be classified as a high leaching additive for these metals (Table 1, entry 5). Leaching of Cu is moderate in this case. These findings are in accordance with the application of Gly as a Co chelator for recycling of Co from lithium ion batteries (Nayaka et al. 2016). For comparison, we measured the leaching properties of *N*-methylglycine (Sarcosine, Sar, Table 1, entry 6) and *N*-dimethylglycine (DMG, Table 1, entry 7) and observed decreasing concentrations of dissolved Co and Ni with increasing steric demand of the Gly derivatives. Similar trends were observed with other proteinogenic amino acids (Table 1, entries 8–11). All amino acids tested with either increased steric hindrance through side chains at the α-carbon or the amino group are less corrosive to Co and Ni compared to Gly. It should be noted that a similar effect of sterically demanding sidechains is not observed for Cu leaching. The leaching of Co and Ni is significantly increased for all amino carboxylic acids tested compared to amino alcohols like TEA, MIPA, or AMP. The observed difference in leaching of Co and Ni versus Cu is reflected by the corresponding stability constants of amino carboxylic acid complexes with Co(II), Ni(II), and Cu(II) (Gergely et al. 1972). The reported complex stability constants reveal a small difference in stability for Gly complexes of Cu(II) compared to complexes with sterically more demanding α-amino acids. In contrast, significant differences in complex stability have been reported for Ni(II) and Co(II) complexes of amino acids with different steric demands. For both metals, Gly complexes were found to be the most stable. The observed dependence of complex stability from steric factors is most likely a consequence of a different complex geometry for Co(II), Ni(II), and Cu(II) complexes. Amino acid complexes of Co(II) and Ni(II) have a 3:1 stoichiometry of ligand:metal with an octahedral coordination of the metal by three bidentate α-amino carboxylate ligands (Gu et al. 2007), whereas amino acid complexes of Cu(II) have a 2:1 stoichiometry of ligand:Cu with a planar coordination of the metal by two bidentate α-amino carboxylate ligands (Casari et al. 2004). The latter planar arrangement is less crowded and therefore less sensitive to the steric impact of bulky side chains. Of all amino acids tested, Tau and AHX stand out with respect to their low corrosivity toward Co, Ni, and Cu, while the α-amino sulfonic acid Tau (Table 1, entry 13) leads to concentrations of dissolved metal in the same range as the benchmark amino alcohol TEA, AHX as a mid-chain ω-amino carboxylic acid (Table 1, entry 12) leads to extremely low dissolved metal concentrations which were even lower than values obtained for the low leaching additives AMP and MIPA. Thus, both non-proteinogenic amino acids show a drastically reduced corrosivity toward all three metals tested. Again, this decrease in corrosivity (and consequently improved leaching properties) can be explained with the corresponding complex chemistry as both Tau and AHX are less powerful chelate ligands compared to the proteinogenic α-amino acids mentioned before. We have also tested mixtures of various acidic CIs with AHX for leaching properties. All AHX combinations tested resulted in low concentrations of dissolved Co and Ni qualifying AHX as an excellent low leaching additive particularly for applications of MWFs with Co-hardened tools. Moderate corrosivity and accordingly higher concentrations of dissolved metal were observed for Cu only (Table 1, entries 14–18).

### Electrochemical evaluation of amino acids as alkaline additives

We started the electrochemical evaluation of amino acids in hard water (20 dGH) under the same conditions used for the chip filter assay mentioned above. Hard water was used as media, because it imitates media in industrial applications of metal working fluids quite well. In follow-up experiments, we have also extended our measurements to more corrosive media (aqueous NaCl solutions) as outlined below. S235JR steel coupons were used as test specimens (S235JR steel is a common construction steel used in many industrial processes). All experiments were run at room temperature only to keep the number of experiments manageable. Potentiodynamic polarization measurements were taken after 24 h exposure of S235JR steel coupons to reflect the steady-state conditions after layer formation of the acidic corrosion inhibitors used (Felhősi et al. 2002). Tricarboxylic acid **2** was chosen as a reference for an efficient acidic CI. The combination of **2** with TEA as an alkaline additive is an industrial benchmark system for corrosion protection of iron and steel in hard water. The anti-corrosive properties of this mixture are thus excellent and we have not been able to determine the limiting effective concentration of **2**/TEA in hard water by electrochemical methods. We observed almost perfect protection even at low millimolar concentrations (Table 2, entry 3) as reflected by a low corrosion rate and a high inhibition efficiency of 99.85%. Lower concentrations of **2** lead to a conductivity of the hard water solution too low to obtain reproducible potentiodynamic results. The electrochemical evaluation is thus less sensitive to corrosion compared to the chip filter assay. An important difference is the relatively small and more homogenous surface area exposed to the corrosive media in the electrochemical setup, where a polished bulk metal serves as a working electrode. Thus, even the lowest concentrations of the acidic CI **2** tested were likely far above the limiting effective concentration for complete corrosion protection. The choice of alkaline additive has therefore almost no influence on the protective effect of CI **2** (at least during the measurement time span of 24 h). Even neutralization of CI **2** with NaOH leads to perfect corrosion inhibition (Table 2, entry 2). The same perfect anti-corrosive effect was observed for the addition of sodium glutamate (Table 2, entry 4), confirming the finding that glutamate as a substitute of TEA has at least no negative effect on the anti-corrosive properties of CI **2**. Compared to TEA, Glu might even improve the protective properties of **2** slightly as reflected by a more positive value for the polarization resistance (Table 2, entry 4). More pronounced was the influence of the alkaline additive on the carboxyphosphonic acid **3**. We have previously reported, that **3** is a less powerful CI in hard water solution than **2** and attributed this loss of performance to the low hard water compatibility of carboxyphosphonic acid **3** leading to precipitation of hardly soluble salts with di- and trivalent cations (Ruf et al. 2022). Carboxyphosphonic acid **3** is thus less effective in corrosion protection of steel when neutralized with TEA compared to carboxylic acid **2** (Table 2, entry 5). The time-dependent OCP in Fig. 2A reveals a slow immobilization of **3** on steel accompanied by a steady decrease in potential value, which is characteristic for the self-assembly of protective nanolayers by most amphiphilic phosphonic acids (Felhősi et al. 2002). However, after 16 h, a rapid increase in the value of potential is observed accounting for the partial degradation of the protecting layer on the metal surface. In parallel, a milky suspension is formed indicating the precipitation of phosphonate salts. When TEA was substituted by sodium glutamate for neutralization of CI **3**, we observed a decrease in value of OCP over several hours down to a constant value indicating the formation of stable protective layers (Fig. 2A). The potentiodynamic curve reveals a pronounced anodic shift of the corrosion potential (Fig. 2B). All electrochemical parameters calculated from the potentiodynamic measurements correspond to almost complete corrosion protection of steel for **3**/Glu (Table 2, entry 6), and we determined an extremely low corrosion rate of 0.15 µm/y and a high positive value for the polarization resistance. Electrochemical impedance measurements confirm these findings. Bode plots of electrochemical impedance measurements show the high anti-corrosive efficiency of tricarboxylic acid **2** which is independent from the additives NaOH, TEA, or Glu under the conditions employed. For carboxyphosphonic acid **3**, in contrast, the alkaline additive has an influence on the formation of protective layers and thus anti-corrosive efficacy. The values of the Bode modules in the low-frequency region are higher for **3**/Glu compared to **3**/TEA (Fig. 2C). The corresponding capacitive loop in the Nyquist plots is also larger (Fig. 2D). Both, high Bode moduli and large capacitive loops in Nyquist plots correlate with high corrosion resistance (Mansfeld 1990). We observed still a slightly milky suspension for **3**/Glu; however, significantly less precipitate was formed (compared to **3**/TEA) suggesting that Glu improves the hard water compatibility of carboxyphosphonic acid **3** and serves as an anti-scalant, similarly to other poly(carboxylic) acids (Jafar Mazumder 2020).

**Table 2:** Results of the potentiodynamic polarization measurements of S235JR-Steel after 24 h exposure against hard water solution (20 dGH, pH 8.3 ± 0.5) containing tricarboxylic acid **2** or carboxyphosphonic acid **3** and various alkaline additives at room temperature

Entry	CI, c (mmol/L)	Additive	*i*_corr_ (nA/cm^2^)	IE_Icorr_ (%)	*E*_corr_ (mV)	*β*_*a*_ (mV/dec)	*β*_*c*_ (mV/dec)	*CR* (µm/y)	*R*_*p*_ (MΩ)
1	–	NaOH^a^	37,500	–	− 584	544	1540	436	0.003
2	**2**, 2.2	NaOH^b^	43.8	99.89	− 123	156	79.7	0.35	5.464
3	**2**, 2.2	TEA^b^	56.2	99.85	− 141	184	76.9	0.65	0.416
4	**2**, 2.2	Glu^b^	12.5	99.97	− 153	171	76.5	0.14	2.663
5	**3**, 18.8	TEA^b^	1790	95.23	− 368	402	244	20.8	0.032
6	**3**, 18.8	Glu^b^	5.72	99.98	− 142	186	61.5	0.15	4.523

^a^pH was adjusted with 1 M NaOH to 8.0

^b^4.5 molar equivalents were used. Glu was used as disodium salt

The observed antiscaling effect of Glu with carboxyphosphonic acid **3** in hard water was interesting to note. However, it was difficult to derive further information regarding synergistic additive effects from the electrochemical experiments in hard water because all mixtures tested revealed almost perfect corrosion protection under these conditions. We changed the media therefore to more corrosive aqueous 0.5% NaCl solution. Carboxyphosphonic acids like **3** or **4** have been shown to have good anti-corrosive properties in chloride containing media. Both compounds were therefore selected as acidic CIs and evaluated in combination with NaOH, TEA, and different amino acid salts as alkaline additives. We determined the limiting effective concentrations of these combinations (with IE_*I*corr_ > 99%) to achieve complete corrosion protection by potentiodynamic measurements, and a selection of data is presented in Tables 3 and 4. The limiting effective concentration for **4**/NaOH was found to be around 30–35 mmol/L (Table 3, entries 2–4) as reflected by IE_*I*corr_ > 99%, low corrosion rates and a high positive value of polarization resistance. The limiting effective concentration for **3**/NaOH is slightly higher at 35–40 mmol/L (Table 4, entries 2–3). The substitution of NaOH by TEA decreased the corrosion rate of carboxyphosphonic acid **4** significantly (Table 3 entries 5–6), leading to an effective limiting concentration of 20 mmol/L. In contrast, substitution of NaOH by TEA had almost no effect for carboxyphosphonic acid **3** and the limiting effective concentration remained at 35–40 mmol/L for **3**/TEA (Table 4, entries 4–5). The combination of carboxyphosphonic acid **3** with Tau as an alkaline additive improved the anti-corrosive properties of **3** to a low effective limiting concentration of 20–25 mmol/L (Table 4, entries 6–7). Tau shows thus the most promising properties as an alkaline additive for **3** in potentiodynamic studies. This finding is supported by high values of polarization resistance *R*_*p*_ and high impedance values at lower frequencies in the Bode plots. The addition of AHX leads to a less-effective corrosion protection with CI **3** reflected by a higher effective limiting concentration of 60–70 mmol/L. The typical layer forming protective mechanism of amphiphilic phosphonic acids on steel is reflected by a steady decrease in OCP value over several hours as observed for **3**/NaOH (Fig. 4A). Similar time-dependent OCPs were observed for **3**/Tau and with slightly higher values also for **3**/TEA. For **3**/AHX, however, OCPs are significantly higher in value suggesting a less perfect layer formation. It is interesting to note, that the mixture of **3**/AHX has a significantly lower critical micelle concentration (cmc) of 1.7 mmol/L (measured by ^1^H-NMR, see supporting information, Table S1) compared to **3**/NaOH (30 mmol/L). Although not strictly comparable with the formation of protective monolayers on steel, this decreased cmc suggests a higher surface activity for **3**/AHX compared to **3**/NaOH which should come with better corrosion protection. The opposite effect was observed and other factors besides surface activity must be operating. Bode plots of electrochemical impedance measurements confirm the high efficiency of **3**/NaOH, **3**/TEA, and **3**/Tau as layer-forming CIs, because the values of the Bode modules in the low-frequency region are highest for these three mixtures (Fig. 4C). Their capacitive loops in the Nyquist plots are also the largest among the mixtures tested (Fig. 4D). As mentioned above, both high Bode moduli and large capacitive loops in Nyquist plots correlate with high corrosion resistance (Mansfeld 1990). Bode plots of all mixtures including carboxyphosphonic acid **3** reveal only one time constant, suggesting charge-transfer-controlled corrosion processes (Macdonald 2005). A comparison with the second carboxyphosphonic acid **4** reveals a different influence of the alkaline additive. All amino acid additives lead to a significant decrease of limiting effective concentrations compared to **4**/NaOH (Table 3). Again, AHX and also Gly were least effective and gave the highest limiting concentrations for the corresponding mixtures with **4**, whereas Tau and Glu had the best effect on corrosion protection of **4** and gave the lowest limiting concentrations (Table 3, entries 7–10) as reflected by all electrochemical parameters, such as *IE*_*Icorr*_ > 99%, low corrosion rate, and high positive values, for resistance polarization. The corresponding time-dependent OCPs (all measured at a concentration of 20 mmol/L for CI **4**) confirm the formation of protective layers with **4**/Tau, **4**/Glu, and **4**/TEA with a regular decrease of OCP values within a few hours to a low constant value around − 100 to − 200 mV. For **4**/AHX and **4**/NaOH, significantly higher OCP values (− 300 mV to − 400 mV) were observed suggesting the formation of less perfect protective layers on steel. The potentiodynamic curves shown in Fig. 3B confirm the trend in efficacy for the alkaline additives tested and their pronounced influence on the anodic corrosion reaction. The noblest potentials and lowest current densities were observed for **4**/Tau and **4**/Glu followed by **4**/TEA, **4**/AHX and **4**/NaOH. The curves are significantly shifted in this series, suggesting a pronounced influence of the alkaline additive on the anti-corrosive properties of **4**. We have again measured the influence of the additive on the cmc of **4** in water by ^1^H-NMR, but observed only small differences (cmc_**4**/NaOH_ = 4.5 mmol/L, cmc_**4**/TEA_ = 4.7 mmol/L, cmc_**4**/AHX_ = 7.8 mmol/L) which can again not account for the observed large differences in anti-corrosive properties. Instead we assume, that amino acids are surface-active by creating active sites on the metal surface, presumably by a fast interaction with hydrated iron oxide films and create thus favorable anchoring points for the stable immobilization of the acidic CIs through chemisorption. This hypothesis is supported by the fact, that the largest synergistic effects were found for amphiphilic phosphonic acids like **4** with a relatively slow immobilization on metal surfaces. Amino acids might thus have similar properties to other surface-active alkaline additives (Felhősi et al. 2002; Kern and Landolt 2001; Ochoa et al. 2005).

**Table 3:** Results of the potentiodynamic polarization measurements of S235JR steel after 24 h exposure to aqueous NaCl solution (0.5wt.%, pH 8.3 ± 0.5) containing carboxyphosphonic acid **4** and various alkaline additives at room temperature

Entry	**4**, (mmol/L)	Additive	*i*_corr_ (nA/cm^2^)	IE_Icorr_ (%)	*E*_corr_ (mV)	*β*_*a*_ (mV/dec)	*β*_*c*_ (mV/dec)	*CR* (µm /y)	*R*_*p*_ (MΩ)
1	–	NaOH^a^	26,900	–	− 511	136	144,600	313	0.003
2	20	NaOH^a^	19,200	28.62	− 430	235	1020	223	0.004
3	30	NaOH^a^	536	98.01	− 182	260	102	62.3	0.072
4	35	NaOH^a^	9.51	99.96	− 153	251	79.1	0.111	2.132
5	15	TEA^b^	–^c^	–^c^	–^c^	–^c^	–^c^	–^c^	0.161
6	20	TEA^b^	11.4	99.96	− 190	382	37.9	0.134	0.687
7	15^a^	Glu^b^	14,800	44.98	− 458	193	460	167	0.004
8	20^a^	Glu^b^	7.59	99.97	− 116	237	69.5	0.092	2.882
9	10^a^	Tau^b^	10,800	59.85	− 314	269	272	125	0.005
10	15^a^	Tau^b^	3.80	99.97	− 115	173	68.0	0.044	2.785
11	25^a^	AHX^b^	3030	88.74	− 413	344	166	35.2	0.015
12	30^a^	AHX^b^	27.4	99.90	− 221	476	87.7	0.032	1.026
13	35^a^	AHX^b^	3.93	99.99	− 113	177	67.7	0.046	2.338

^a^pH was adjusted with 1 M NaOH to 8.0

^b^4.5 molar equivalents were used. Amino acids were used as sodium salts

^c^Inconclusive Tafel-Plot due to extensive pitting corrosion

**Table 4:** Results of the potentiodynamic polarization measurements of S235JR steel after 24 h exposure to aqueous NaCl solution (0.5wt.%, pH 8.3 ± 0.5) containing carboxyphosphonic acid **3** and various alkaline additives at room temperature

Entry	**3**, (mmol/L)	Additive	*i*_corr_ (nA/cm^2^)	IE_Icorr_ (%)	E_corr_ (mV)	*β*_*a*_ (mV/dec)	*β*_*c*_ (mV/dec)	*CR* (µm/y)	*R*_*p*_ (MΩ)
1	30	NaOH^a^	365	98.64	− 296	978	93.5	4.18	0.089
2	35	NaOH^a^	364	98.65	− 258	518	87.5	4.23	0.084
3	40	NaOH^a^	14.1	99.95	− 164	183	71.7	0.164	1.201
4	35	TEA^b^	506	98.12	− 581	38.9	106	5.88	0.036
5	40	TEA^b^	37.5	99.86	− 240	263	68.2	0.436	0.507
6	20	Tau^b^	10,000	32.89	− 347	242	340	85.3	0.008
7	25	Tau^b^	10.9	99.94	− 124	518	76.0	0.126	8.122
8	40	AHX^b^	1.420	94.57	− 322	757	129	16.4	0.025
9	60	AHX^b^	1.470	94.54	− 305	1270	146	17.1	0.037
10	70	AHX^b^	253	99.06	− 297	802	115	11.1	0.068

^a^pH was adjusted with 1 M NaOH to 8.0

^b^4.5 molar equivalents were used. Amino acids were used as sodium salts

### Gravimetric evaluation of anti-corrosive properties

**Fig. 5:**
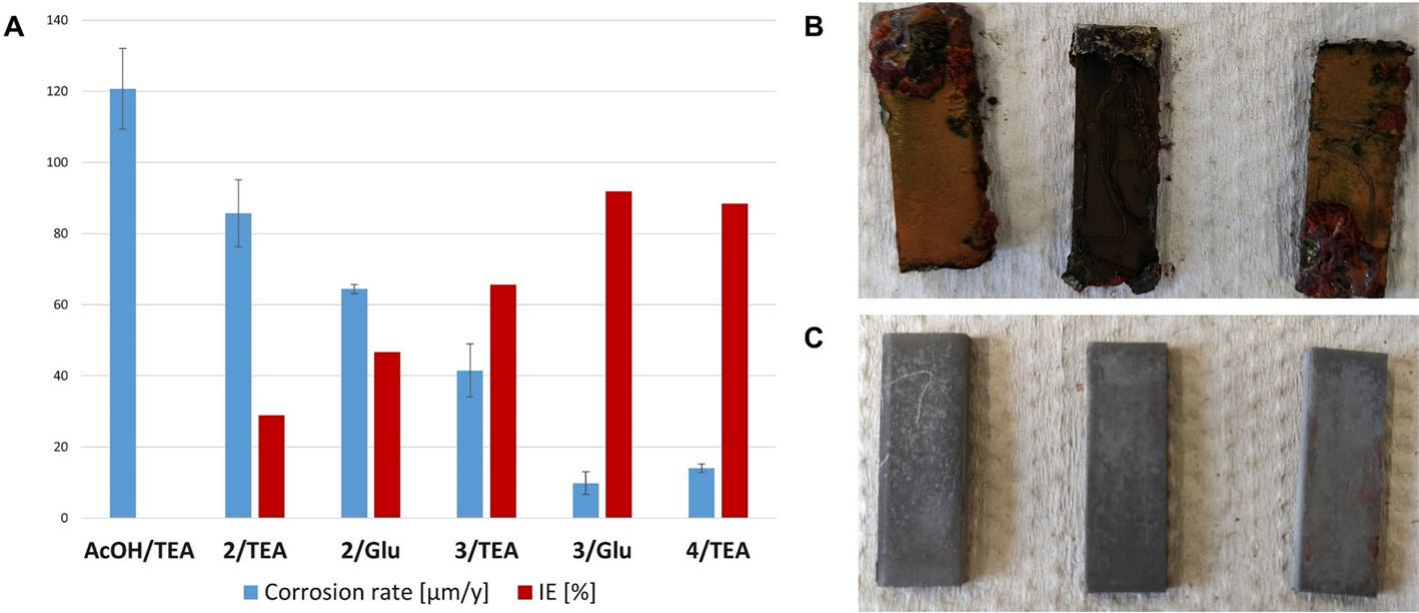
**A** Results of the gravimetric tests on S235JR. Rectangular steel slides (30 × 10 × 3 mm) were immersed in test solutions containing 3wt% of the acidic CI for 12 weeks at rt. 1.5 molar equivalents of an alkaline additive for each acidic proton in acidic CIs were added. Medium: aqueous 2wt.% NaCl solution (pH 8.3 ± 0.5). IE was calculated for each CI in relation to the non-inhibited experiment (AcOH/TEA buffer). **B** Staining of steel slides after 12 weeks exposure to aqueous **2**/TEA and **C** **2**/Glu

As noted previously, the performance of layer-forming CIs can be time-dependent. Since many industrial applications require efficient corrosion inhibition over longer periods than those measured with our electrochemical studies, we included a long-term gravimetric assay with rectangular steel coupons (S235JR) of 30 × 10 × 3 mm size. Due to the choice of test slides (S235JR) with intrinsically high corrosion resistance and the limited sensitivity of gravimetric measurements, corrosive media were needed to increase the weight loss upon corrosion of test coupons to reliably detectable amounts within a reasonable timespan. When 0.5% aqueous NaCl was used as media (as in the electrochemical experiments), only low mass losses even for the non-inhibited solutions were observed leading to low reproducibility of the measurements. 2% aqueous NaCl was therefore used as media, sufficiently corrosive to provide a weight loss of 186 mg within 12 weeks of incubation at room temperature and constant pH ~ 8 (TEA/AcOH buffer) when no additional CI was added. This amounts to a corrosion rate of 121 µm/y (Fig. 5A). All CIs tested were compared to this value, and the corresponding reduction in corrosion is given in Fig. 5A (red bars, inhibitory efficiency, IE) next to the corrosion rates (blue bars). We have used the TEA/AcOH buffer as a reference system, because the pH is in the same range as in our test solutions, and AcOH is known to have almost no anti-corrosive properties at neutral or slightly alkaline pH values on iron and steel (Hefter et al. 1997). We have previously reported, that **2**/TEA does not show good corrosion protection in chloride containing media (IE = 29%), whereas **3**/TEA is moderately effective (IE = 66%) and **4**/TEA is highly effective under these conditions (IE = 88%). The use of amino acid salts such as Glu instead of TEA improves the anti-corrosive properties of **2** and **3** in NaCl solution significantly and leads to almost perfect corrosion inhibition with **3**/Glu (IE = 92%). With **2**, corrosion is reduced to a less perfect (IE = 47%) but still improved value, when Glu was used as an alkaline additive instead of TEA. However, visual inspection of the test specimens after incubation in 2% aqueous NaCl for 12 weeks revealed almost polished metal surfaces for **2**/Glu (Fig. 5C), whereas large quantities of corrosion products were deposited for **2**/TEA (Fig. 5B). As noted above, Glu serves as an antiscalant preventing the deposition of corrosion products on the metal surface.

## Conclusions

A set of proteinogenic and non-proteinogenic amino acids was evaluated for their potential as alkaline additives to acidic CIs for application in aqueous MWFs of slightly alkaline pH. Amino acids were employed as sodium salts and used for neutralization of common acidic CIs of the carboxylic acid and the phosphonic acid type. The resulting mixtures were analyzed with respect to Co, Ni, and Cu leaching and anti-corrosive properties for iron and steel. Major findings are:

1. All α-amino carboxylic acids lead to relatively strong leaching of alloying metals, such as Co and Ni. An extraction assay revealed that Gly addition gave the highest concentrations of Co and Ni in solution. Leaching of Co and Ni decreases with increasing steric demand of substituents in α-amino carboxylic acids. However, all α-amino carboxylic acids tested lead to higher leaching of alloying metals than industrial low leaching amino alcohols like MIPA or AMP. The leaching properties of alkaline additives depend on the corresponding complex stabilities and Tau as well as AHX, which are both bad chelators, lead to low leaching of Co and Ni. Particularly AHX is an attractive low leaching additive leading to lower Co and Ni concentrations in solution than MIPA and AMP.
2. A visual chip filter assay of various mixtures of amino acids and acidic CIs revealed that many amino acids are compatible with CIs of the carboxylic and phosphonic acid type. Synergistic effects on the anti-corrosive properties of CIs were observed for Met, Glu, Tau, and AHX. However, Met was excluded from more detailed analyses, because of the limited chemical stability of the solutions.
3. Electrochemical evaluation of Glu, Tau, and AHX as additives to a tricarboxylic acid (**2**) and two carboxyphosphonic acids (**3**) and (**4**) confirmed synergistic anti-corrosive effects of Glu and Tau. Impedance measurements and time-dependent OCPs showed that Glu and Tau improve the layer-forming anti-corrosive mechanism of the CIs tested. The strongest anti-corrosive effect was observed for Tau/**4**. We assume that amino acid salts interact fast with hydrated iron oxide films and create thus favorable anchoring points for the stable but slow immobilization of the acidic CIs through chemisorption. In addition, Glu improved the hard water compatibility of phosphonic acid **3** and is thus increasing its anti-corrosive properties in hard water significantly. In summary, Glu and Tau improved the anti-corrosive properties of all CIs tested and let to almost perfect corrosion inhibition at low millimolar concentrations of CIs even in corrosive media like 0.5wt.% aqueous NaCl. Mixtures of acidic CIs with Glu and Tau were thus significantly more efficient than mixtures with the amino alcohol TEA.
4. The antiscaling activity and the synergistic anti-corrosive effect of Glu were also confirmed by long-term gravimetric tests on steel with carboxylic acid **2** and carboxyphosphonic acid **3**.

In summary, sodium salts of amino acids are valuable alkaline additives for neutralization of acidic CIs. Glu and Tau had a synergistic anti-corrosive effect on several acidic corrosion inhibitors and were thus superior to common additives like TEA. Several amino acids have an added value, such as antiscaling properties (Glu) or low leaching of Co and Ni (Tau and AHX). Amino acid salts may thus be used as economically and ecologically attractive substitutes for currently used amino alcohols.

### Patents

As a result of the work reported herein, a patent application has been filed: EP/2021/053871, “Amino acids as green neutralizing agents for acidic corrosion inhibitors”.

## Supplementary Information

Below is the link to the electronic supplementary material.

Supplementary file1 (DOCX 2865 KB)

## Data Availability

The data that support the findings of this study are available from the corresponding author upon request.

## References

[CR1] Abohalkuma T, Shaban A, Telegdi J (2016). Corrosion processes controlled by phosphonic acid nano-layers. Period Polytech-Chem.

[CR2] Anastas PT, Kirchhoff MM (2002). Origins, current status, and future challenges of green chemistry. Acc Chem Res.

[CR3] Barceloux DG (1999). Cobalt. J Toxicol Clin Toxicol.

[CR4] Bay N, Azushima A, Groche P (2010). Environmentally benign tribo-systems for metal forming. CIRP Ann.

[CR5] Beerthuis R, Rothenberg G, Shiju NR (2015). Catalytic routes towards acrylic acid, adipic acid and ε-caprolactam starting from biorenewables. Green Chem.

[CR6] Bennett EO, Bennett DL (1984). Metalworking fluids and nitrosamines. Tribol Int.

[CR7] Brenna A, Bolzoni F, Pedeferri MP, Ormellese M (2017). Corrosion inhibitors for reinforced concrete structures: a study of binary mixtures. Int J Corros Scale Inhib.

[CR8] Brinksmeier E, Meyer D, Huesmann-Cordes AG, Herrmann C (2015). Metalworking fluids—mechanisms and performance. CIRP Ann.

[CR9] Bruze M, Hradil E, Eriksohn I-L, Gruvberger B, Widström L (1995). Occupational allergic contact dermatitis from alkanolamineborates in metalworking fluids. Contact Dermatitis.

[CR10] Byers JP, Byers JP (2017) Metalworking fluids, 3rd edn.

[CR11] Casari BM, Mahmoudkhani AH, Langer V (2004). A redetermination of cis-aquabis(glycinato-[kappa]2N, O)copper(II). Acta Crystallographica Section E.

[CR12] Chu C-Y, Sun C-C (2001). Allergic contact dermatitis from triethanolamine in a sunscreen. Contact Dermatitis.

[CR13] Felhősi I, Telegdi J, Pálinkás G, Kálmán E (2002). Kinetics of self-assembled layer formation on iron. Electrochim Acta.

[CR14] Fiume MM, Heldreth B, Bergfeld WF (2013). Safety assessment of triethanolamine and triethanolamine-containing ingredients as used in cosmetics. Int J Toxicol.

[CR15] Gergely A, Sóvágó I, Nagypaál I, Király R (1972). Equilibrium relations of alpha-aminoacid mixed complexes of transition metal ions. Inorg Chim Acta.

[CR16] Groysman A (2010) Corrosion for everybody. Corrosion for Everybody. 10.1007/978-90-481-3477-9

[CR17] Gu K-Q, Sun Y-X, Zhang R, Zhang N-W, Che H-W (2007). Tris(glycinato-[kappa]2N, O)cobalt(III). Acta Crystallographica Section E.

[CR18] Hefter GT, North NA, Tan SH (1997). Organic corrosion inhibitors in neutral solutions; part 1—inhibition of steel, copper, and aluminum by straight chain carboxylates. Corrosion.

[CR19] Jafar Mazumder MA (2020). A review of green scale inhibitors: process, types, mechanism and properties. Coatings.

[CR20] Jamil HE, Montemor MF, Boulif R, Shriri A, Ferreira MGS (2003). An electrochemical and analytical approach to the inhibition mechanism of an amino-alcohol-based corrosion inhibitor for reinforced concrete. Electrochim Acta.

[CR21] Kasprzhitskii A, Lazorenko G, Nazdracheva T, Yavna V (2021). Comparative computational study of L-amino acids as green corrosion inhibitors for mild steel. Computation.

[CR22] Kern P, Landolt D (2001) Adsorption of organic corrosion inhibitors on iron in the active and passive state. A replacement reaction between inhibitor and water studied with the rotating quartz crystal microbalance. Electrochimica Acta 47(4):589–598. 10.1016/S0013-4686(01)00781-2

[CR23] Kümmerer K (2007). Sustainable from the very beginning: rational design of molecules by life cycle engineering as an important approach for green pharmacy and green chemistry. Green Chem.

[CR24] Kuznetsov YI (2016) Organic corrosion inhibitors: where are we now? A review. Part II. Passivation and the role of chemical structure of carboxylates. Int J Corros Scale Inhib 5(4):282–318. 10.17675/2305-6894-2016-5-4-1

[CR25] León González, Rodríguez Gómez FJ, Espinoza Vázquez A JP (2021). Electrochemical behaviour of some amino acids as corrosion inhibitors for mild steel in sweet brine. SOJ Mater Sci Eng.

[CR26] Libralato G, Volpi Ghirardini A, Avezzù F (2010). Seawater ecotoxicity of monoethanolamine, diethanolamine and triethanolamine. J Hazard Mater.

[CR27] Lotierzo A, Pifferi V, Ardizzone S, Pasqualin P, Cappelletti G (2016). Insight into the role of amines in Metal Working Fluids. Corros Sci.

[CR28] Macdonald J (2005). Impedance spectroscopy: models, data fitting, and analysis. Solid State Ionics.

[CR29] Mansfeld F (1990). Electrochemical impedance spectroscopy (EIS) as a new tool for investigating methods of corrosion protection. Electrochim Acta.

[CR30] Mobin M, Parveen M, Rafiquee MZA (2017). Synergistic effect of sodium dodecyl sulfate and cetyltrimethyl ammonium bromide on the corrosion inhibition behavior of l-methionine on mild steel in acidic medium. Arab J Chem.

[CR31] Moschona A, Plesu N, Mezei G, Thomas AG, Demadis KD (2018). Corrosion protection of carbon steel by tetraphosphonates of systematically different molecular size. Corros Sci.

[CR32] Najiha MS, Rahman MM, Yusoff AR (2016). Environmental impacts and hazards associated with metal working fluids and recent advances in the sustainable systems: a review. Renew Sustain Energy Rev.

[CR33] Nayaka GP, Pai KV, Santhosh G, Manjanna J (2016). Recovery of cobalt as cobalt oxalate from spent lithium ion batteries by using glycine as leaching agent. J Environ Chem Eng.

[CR34] Ochoa N, Moran F, Pébère N (2004). The synergistic effect between phosphonocarboxylic acid salts and fatty amines for the corrosion protection of a carbon steel. J Appl Electrochem.

[CR35] Ochoa N, Moran F, Pébère N, Tribollet B (2005). Influence of flow on the corrosion inhibition of carbon steel by fatty amines in association with phosphonocarboxylic acid salts. Corros Sci.

[CR36] Ormellese M, Lazzari L, Goidanich S, Fumagalli G, Brenna A (2009). A study of organic substances as inhibitors for chloride-induced corrosion in concrete. Corros Sci.

[CR37] Pech-Canul MA, Chi-Canul LP (1999). Investigation of the inhibitive effect of *N*-phosphono-methyl-glycine on the corrosion of carbon steel in neutral solutions by electrochemical techniques. Corrosion.

[CR38] Rudnick LR (2005). Synthetics, mineral oils, and bio-based lubricants. Chem Technol.

[CR39] Ruf E, Naundorf T, Seddig T, Kipphardt H, Maison W (2022). Natural product-derived phosphonic acids as corrosion inhibitors for iron and steel. Molecules.

[CR40] Sanyal B (1981). Organic compounds as corrosion inhibitors in different environments—a review. Prog Org Coat.

[CR41] Shirini F, Daneshvar N (2016). Introduction of taurine (2-aminoethanesulfonic acid) as a green bio-organic catalyst for the promotion of organic reactions under green conditions. RSC Adv.

[CR42] Somorjai GA, Frei H, Park JY (2009). Advancing the frontiers in nanocatalysis, biointerfaces, and renewable energy conversion by innovations of surface techniques. J Am Chem Soc.

[CR43] Soylev TA, Richardson MG (2008). Corrosion inhibitors for steel in concrete: state-of-the-art report. Constr Build Mater.

[CR44] Spikes H (2004). The history and mechanisms of ZDDP. Tribol Lett.

[CR45] Umoren SA, Solomon MM, Obot IB, Suleiman RK (2019). A critical review on the recent studies on plant biomaterials as corrosion inhibitors for industrial metals. J Ind Eng Chem.

[CR46] Verma C, Verma DK, Ebenso EE, Quraishi MA (2018). Sulfur and phosphorus heteroatom-containing compounds as corrosion inhibitors: an overview. Heteroat Chem.

[CR47] Verma C, Ebenso EE, Quraishi MA, Hussain CM (2021). Recent developments in sustainable corrosion inhibitors: design, performance and industrial scale applications. Mater Adv.

[CR48] Vyrides I, Rakanta E, Zafeiropoulou T, Batis G (2013). Efficiency of amino alcohols as corrosion inhibitors in reinforced concrete. Open J Civ Eng.

[CR49] Welle A, Liao JD, Kaiser K, Grunze M, Mader U, Blank N (1997). Interactions of *N*, *N*′-dimethylaminoethanol with steel surfaces in alkaline and chlorine containing solutions. Appl Surf Sci.

[CR50] Yang Q, Sherbahn M, Runge T (2016). Basic amino acids as green catalysts for isomerization of glucose to fructose in water. ACS Sustain Chem Eng.

[CR51] Zhang XL, Jia XM, Lian JX (2010). Study on the mechanism of the cobalt leaching of cemented carbide in triethanolamine solution. Adv Mat Res.

[CR52] Zhang C, Duan H, Zhao J (2016). Synergistic inhibition effect of imidazoline derivative and l-cysteine on carbon steel corrosion in a CO_2_-saturated brine solution. Corros Sci.

