# Supplementary material for: Alkali salts of amino acids as alkaline additives for neutralization of acidic corrosion inhibitors
Source: Amino Acids. 2023 Mar 10;55(5):665–78. doi: 10.1007/s00726-023-03260-x (PMC10247853; doi:10.1007/s00726-023-03260-x)
Supplement: Supplementary file 1 — Supplementary file1 (DOCX 2865 KB) [file 726_2023_3260_MOESM1_ESM.docx]

**Supporting Information**

**Alkali salts of amino acids as alkaline additives for neutralization of acidic corrosion inhibitors**

**Tim Naundorf,^1^ Tom Seddig,^1^ Erik Ruf,^1^ Laurens Ballentin,^1^ Helmut Kipphardt^2^ and Wolfgang Maison^1,*^**

^1^ Department of Chemistry, Universität Hamburg, Bundesstraße 45, 20146 Hamburg, Germany.

^2^ Metall-Chemie Technologies GmbH, Herrengraben 30, 20459 Hamburg, Germany.

Corresponding author: Wolfgang Maison, wolfgang.maison@uni-hamburg.de

Table of contents

[**Overview of structures** 1](#_Toc120612663)

[Fig. S1 Chemical structures of acidic CIs and alkaline additives used in this work 1](#_Toc120612664)

[**CMC Evaluations** 2](#_Toc120612665)

[Experimental procedure 2](#_Toc120612666)

[Table S1 cmc values of phosphonic acids 3 and 4 with different alkaline additives determined at rt via ^1^H-NMR in D_2_O 2](#_Toc120612667)

[**NMR Spectra** 3](#_Toc120612668)

[Fig. S2 ^1^H-NMR spectra of 3 + AHX at 400 MHz in D_2_O at different concentrations 3](#_Toc120612669)

[Fig. S3 ^1^H-NMR spectra of 4 + AHX at 400 MHz in D_2_O at different concentrations 4](#_Toc120612670)

[**Chip filter test** 5](#_Toc120612671)

[Table S2 Conditions and corrosion scores of chip-filter-test (according to DIN 51360-02-A) performed with tricarboxylic acid 2 as a commercial acidic CI and various alkaline additives 5](#_Toc120612672)

[Table S3 Conditions and corrosions scores of chip-filter-tests of various acidic CIs in combination with different alkaline additives; pH 8.3 ± 0.8 6](#_Toc120612673)

[Fig. S4 Comparison of staining between a negative control 5 + Gly (entry 4 in Table S3) and 1 + Gly (entry 1 in Table S3) 6](#_Toc120612674)

[**Nyquist Plots (detailed view)** 7](#_Toc120612675)

[Fig. S5 Nyquist plots of electrochemical impedance measurements in detailed view 7](#_Toc120612676)

[**Example of DRT analysis** 8](#_Toc120612677)

[Fig. S6 Results of the DRT analysis for 4 in 0.5 wt% NaCl solution after 24 h (table 3, entry 4). The analysis points to a system with mainly one R/C element. Further mechanistic investigations with an implementation of an equivalent circuit model will follow. 8](#_Toc120612678)

[**References** 8](#_Toc120612679)

# **Overview of structures**

## Fig. S1 Chemical structures of acidic CIs and alkaline additives used in this work

# **CMC Evaluations**

## Experimental procedure

350 µmol of the acid and 1.5 equivalents of the basic additive per acidic proton of the acid were dissolved in 10 mL of D_2_O in a volumetric flask. If necessary, the pH value was adjusted to 8.5 with NaOD. An NMR sample was prepared. 6 mL of the solution were added to a second volumetric flask and diluted to 10 mL with D_2_O. The process was repeated until 10 samples with the concentrations of 35 mmol/L, 21 mmol/L, 13 mmol/L, 7.6 mmol/L, 4.5 mmol/L, 2.7 mmol/L, 1.6 mmol/L, 1.0 mmol/L, 0.59 mmol/L and 0.35 mmol/L were obtained. A suitable peak of the NMR spectrum that showed a noticeable concentration dependent shift was identified and its shift plotted against the inverse concentration. The inflection point indicating the CMC was determined *via* graphical methods.

## Table S1 cmc values of phosphonic acids 3 and 4 with different alkaline additives determined at rt via ^1^H-NMR in D_2_O

| **Substance** | **cmc/NaOH** | **cmc/TEA** | **cmc/AHX** |
| --- | --- | --- | --- |
| **3** | 30 mmol/L^[a]^ | 19.1 mmol/L^[a]^ | 1.7 mmol/L |
| **4** | 4.5 mmol/L^[a]^ | 4.7 mmol/L^[a]^ | 7.8 mmol/L |

^[a]^ cmc values have been listed for comparison and were reported earlier (Ruf et al. 2022).

# **NMR Spectra**

## Fig. S2 ^1^H-NMR spectra of 3 + AHX at 400 MHz in D_2_O at different concentrations

**
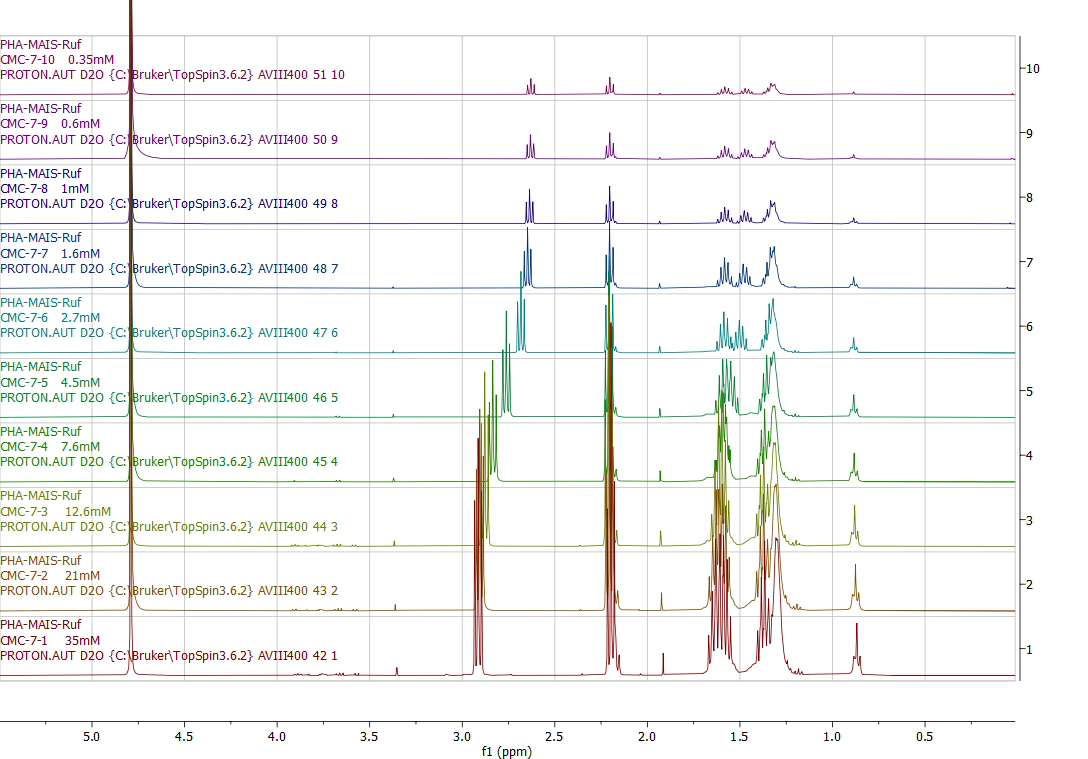
**

## Fig. S3 ^1^H-NMR spectra of 4 + AHX at 400 MHz in D_2_O at different concentrations


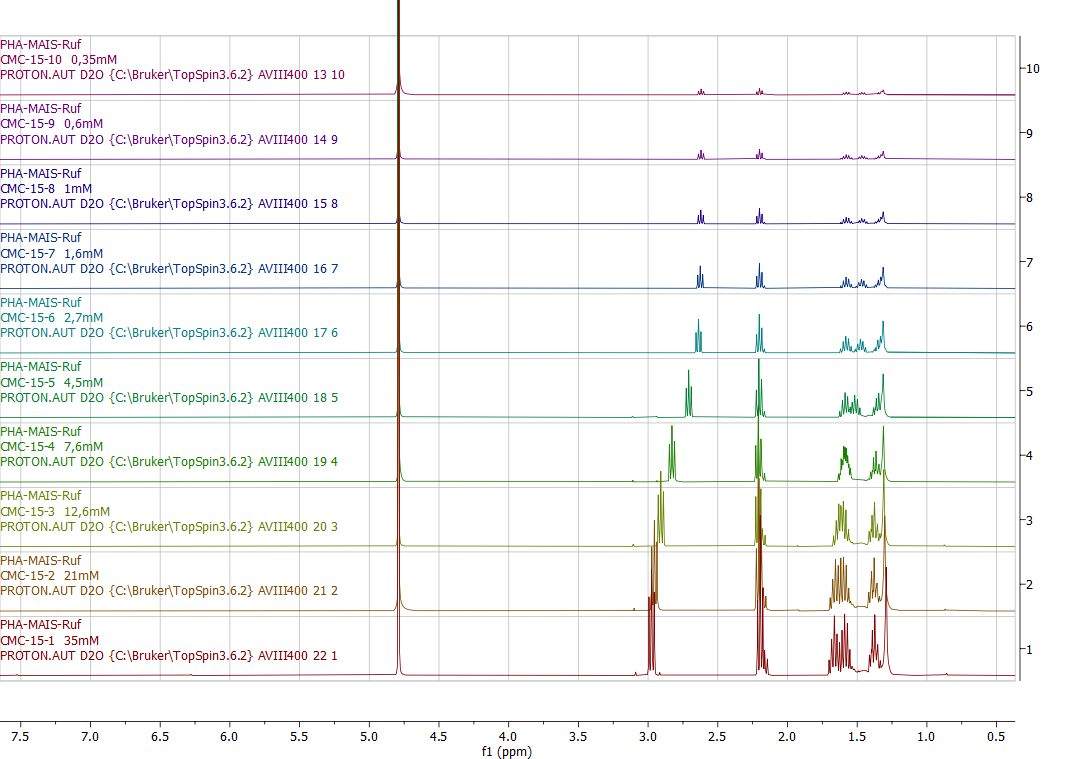


# **Chip filter test**

Evaluation of the anti-corrosive properties of the substances for iron was carried out according to DIN 51360-02-A using the aqueous, solution of the acidic corrosion inhibitors (CIs) and alkaline additive. Grey cast iron turnings on filter paper were treated with test solutions for 2h at room temperature in either hardwater (20 dGH: CaCl_2_ 340 mg/L, MgSO_4_ 60.0 mg/L) or 0.5% aqueous NaCl (pH 8.3 ± 0.5). 1.5 eq alkaline additiv were added for each acidic proton in acidic CIs. As an example, carboxyphosphonic acid **3** contains 3 acidic protons and 4.5 eq TEA were thus added as alkaline additive. 2 mL of the appropriate test solution was incubated with 2.0 g of sieved grey cast iron turnings for 2 h, before rinsing and visual scoring.

## Table S2 Conditions and corrosion scores of chip-filter-test (according to DIN 51360-02-A) performed with tricarboxylic acid 2 as a commercial acidic CI and various alkaline additives

| entry | additive^[a]^ | molar eq | pH | corrosion score |
| --- | --- | --- | --- | --- |
| 1 | TEA | 3.6 | 7.4 | 0 |
| 2 | NaOH | 3.6 | 10.3 | 4 |
| 3 | KOH | 3.6 | 9.1 | 4 |
| 4 | Met | 3.6 | 8.0 | 2 |
| 5 | Gly | 3.6 | 8.8 | 3 |
| 6 | Tau | 3.6 | 8.5 | 1 |
| 7 | Glu | 3.6 | 8.8 | 1 |
| 8 | Ala | 3.6 | 9.0 | 3 |
| 9 | Ser | 3.6 | 8.5 | 1-2 |
| 10 | Arg | 3.6 | 9.0 | 0 |
| 11 | His | 3.6 | 8.5 | 1 |
| 12 | Lys | 3.6 | 9.0 | 4 |
| 13 | AHX | 3.6 | 8.8 | 0 |
| 14 | TEA | 4.5 | 7.8 | 0 |
| 15 | Met | 4.5 | 8.9 | 0-1 |
| 16 | Gly | 4.5 | 9.0 | 1 |
| 17 | Tau | 4.5 | 8.7 | 0-1 |
| 18 | Glu | 4.5 | 9.0 | 1 |
| 19 | AHX | 4.5 | 9.0 | 0 |
| 20 | Gly | 5.0 | 9.0 | 0 |

^[a]^ All aqueous test solutions contained 3wt% tricarboxylic acid **2** as acidic CI and the appropriate alkaline additive as specified in the table. Amino acids were used as sodium salts.

## Table S3 Conditions and corrosions scores of chip-filter-tests of various acidic CIs in combination with different alkaline additives; pH 8.3 ± 0.8

|  |  |  | Corrosion Scores / alkaline additive^[b]^ | | | |  |
| --- | --- | --- | --- | --- | --- | --- | --- |
| entry | CI^[a]^ | molar eq alkaline additive | TEA | Gly | Tau | AHX | |
| 1 | **1** | 3 | 0 | 4(faint) | 0 | 0 | |
| 2 | **4** | 3.6 | 0 | 0 | 0 | 0 | |
| 3 | **4** | 4.5 | 0 | 0 | 0 | 0 | |
| 4 | **5** | 2.5 | 0 | 4(faint) | 0 | 0 | |

^[a]^ All aqueous test solutions contained 3wt% acidic CI. The pH-values after addition of alkaline additives were measured and were pH 8.3 ± 0.8. ^[b]^ Amino acids were used as sodium salts.


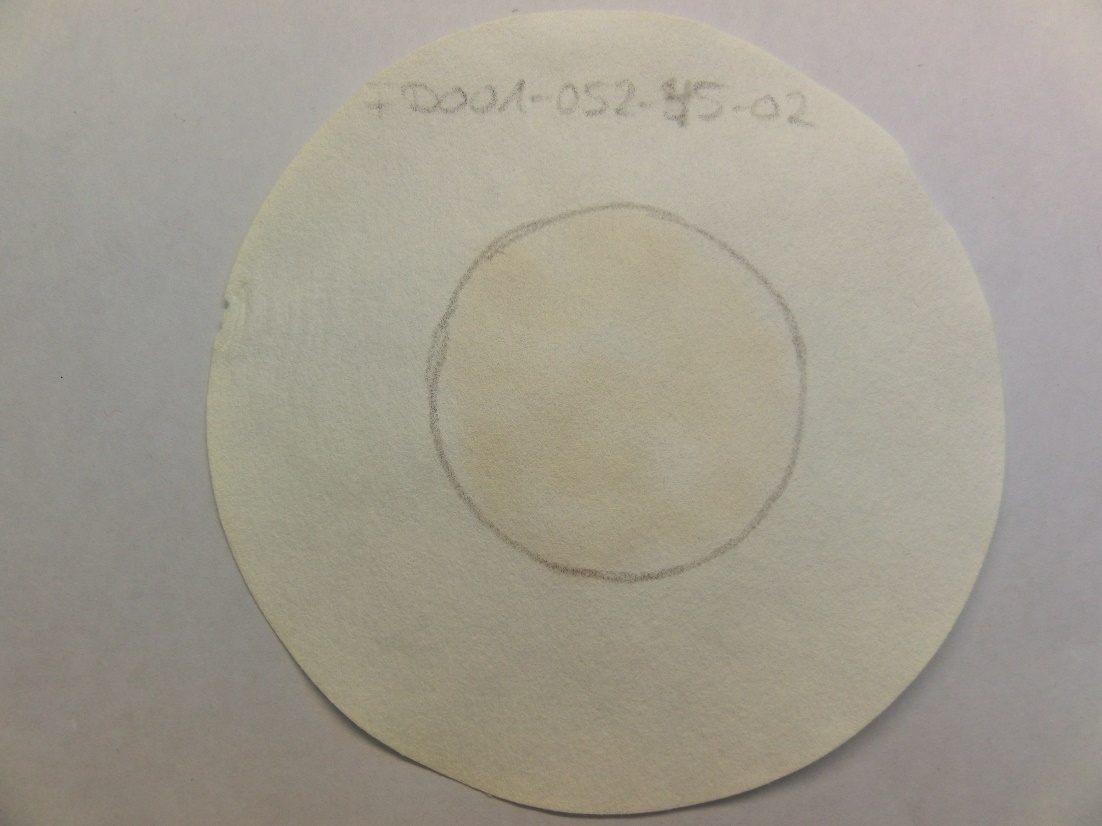

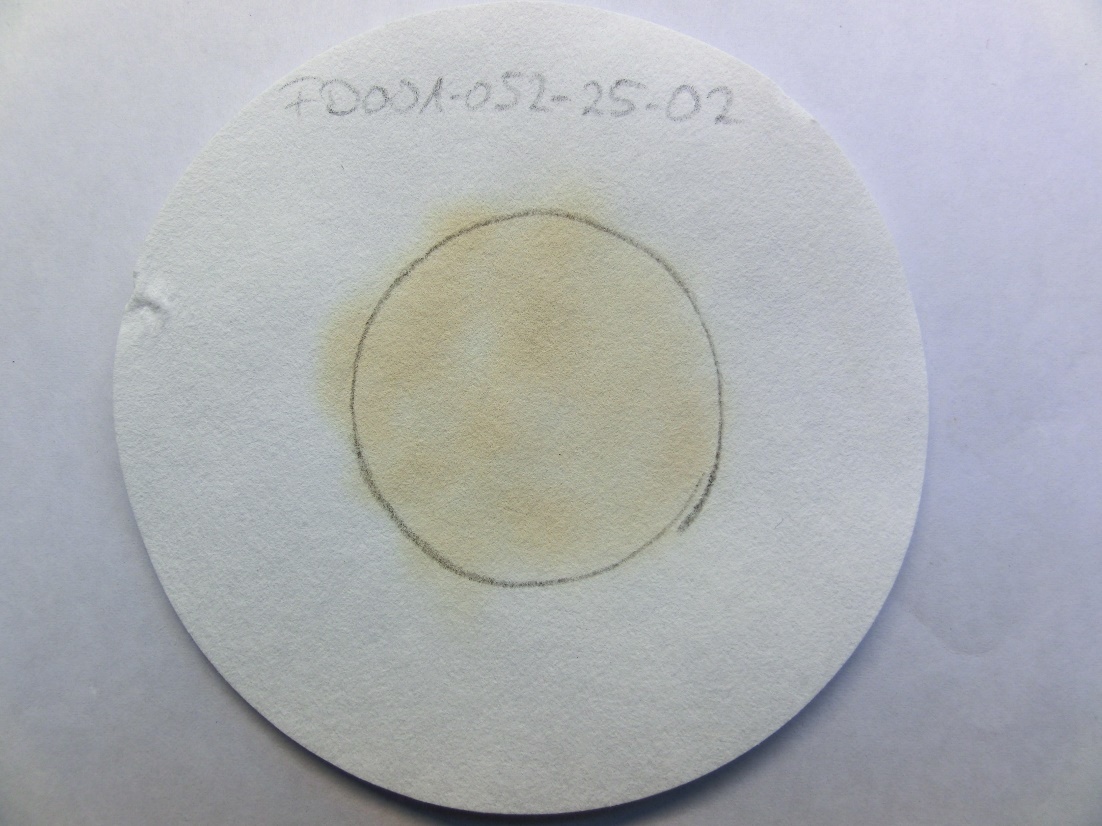

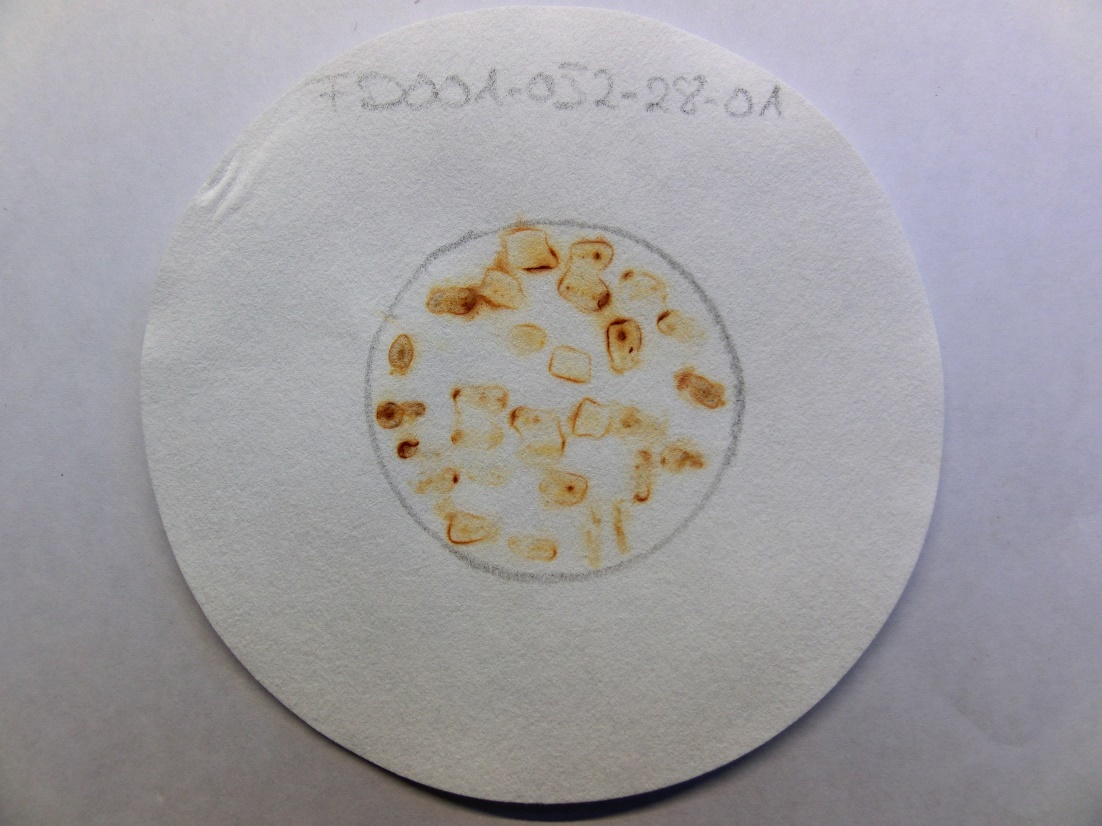


**5** + Gly

**1** + Gly

negative control,

no CI added

## Fig. S4 Comparison of staining between a negative control 5 + Gly (entry 4 in Table S3) and 1 + Gly (entry 1 in Table S3)

# **Nyquist Plots (detailed view)**

| 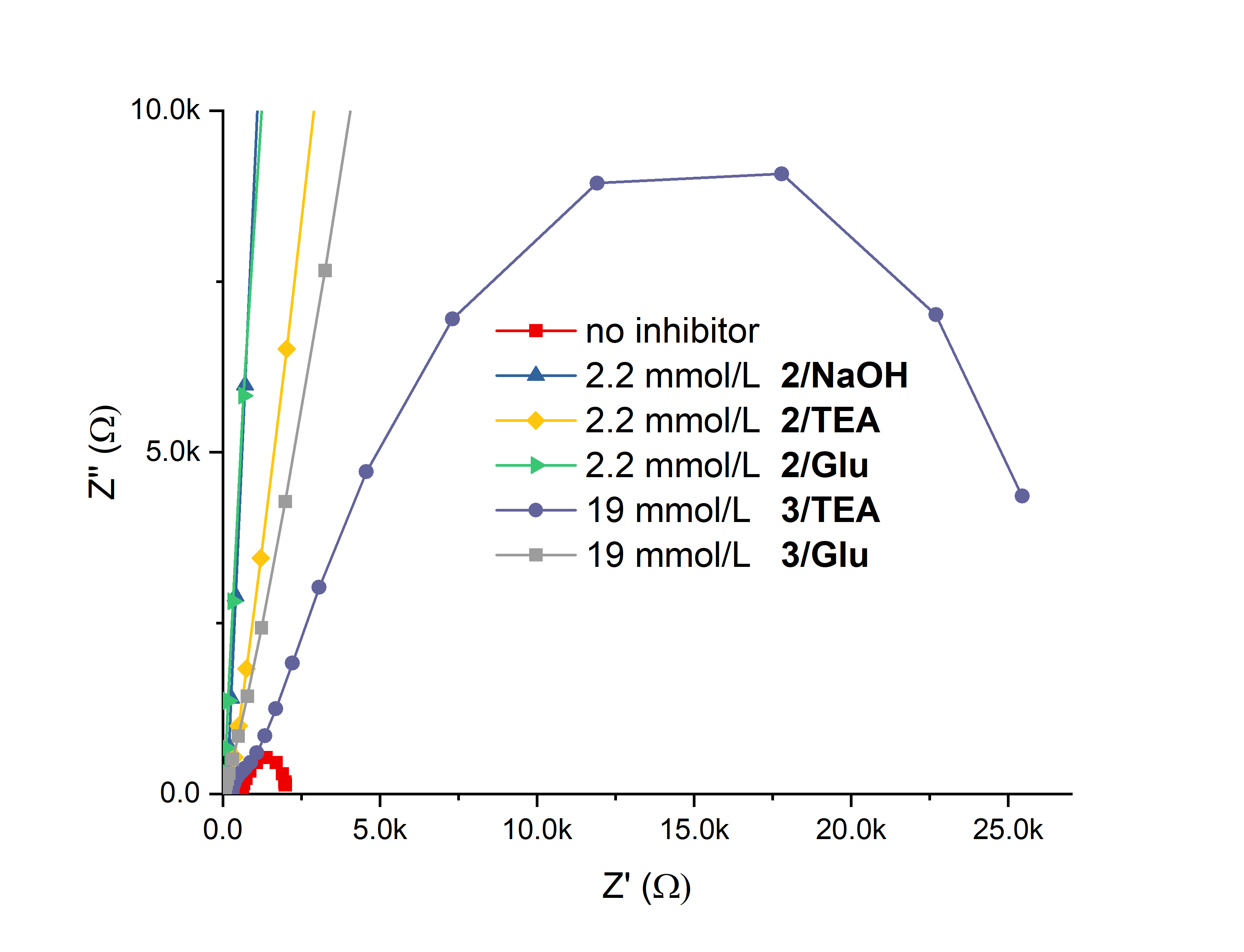 | 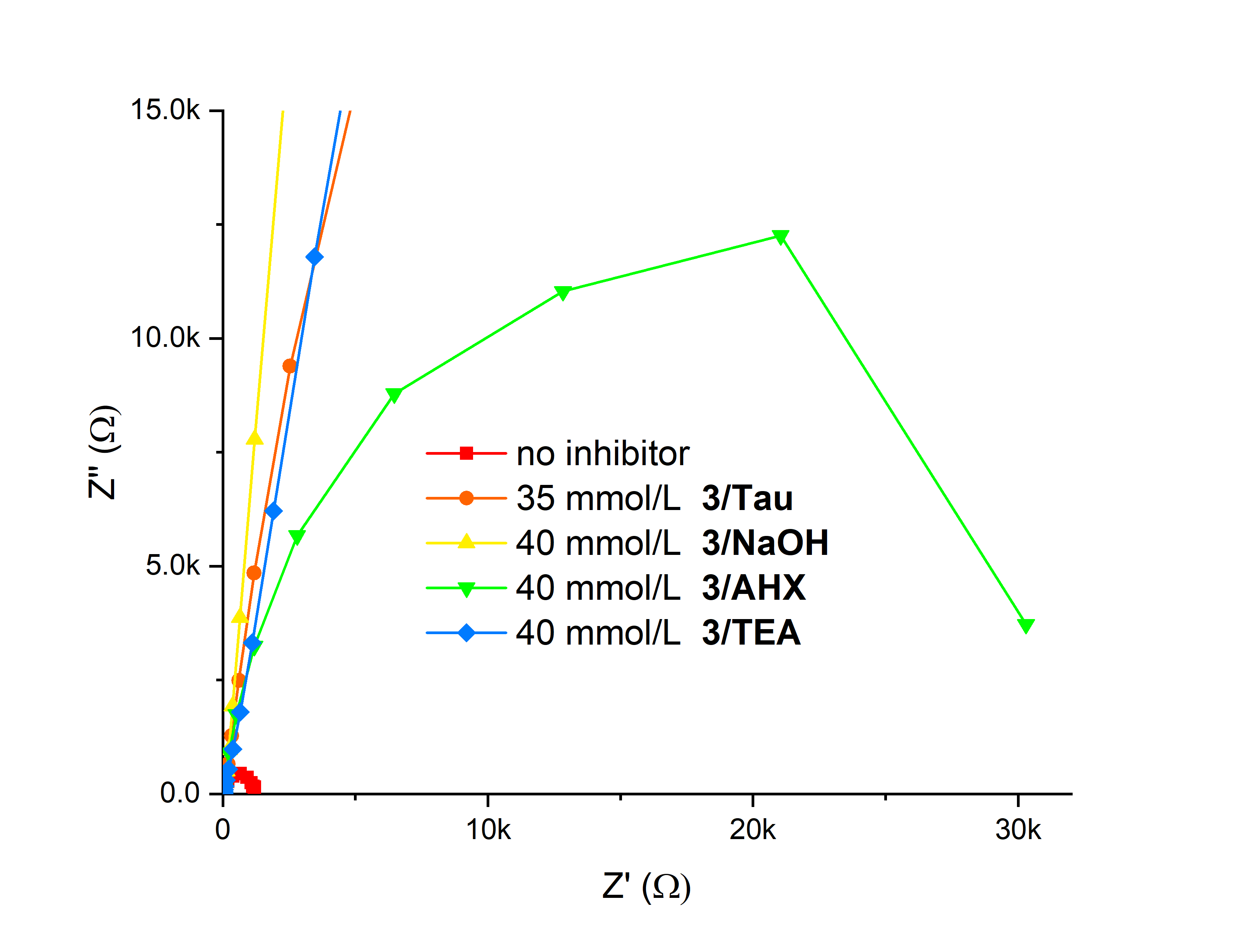 |
| --- | --- |
| hardwater-solution (20 dGH, pH 8.3 ± 0.5) | NaCl-solution (0.5wt.%, pH 8.3 ± 0.5) |
| 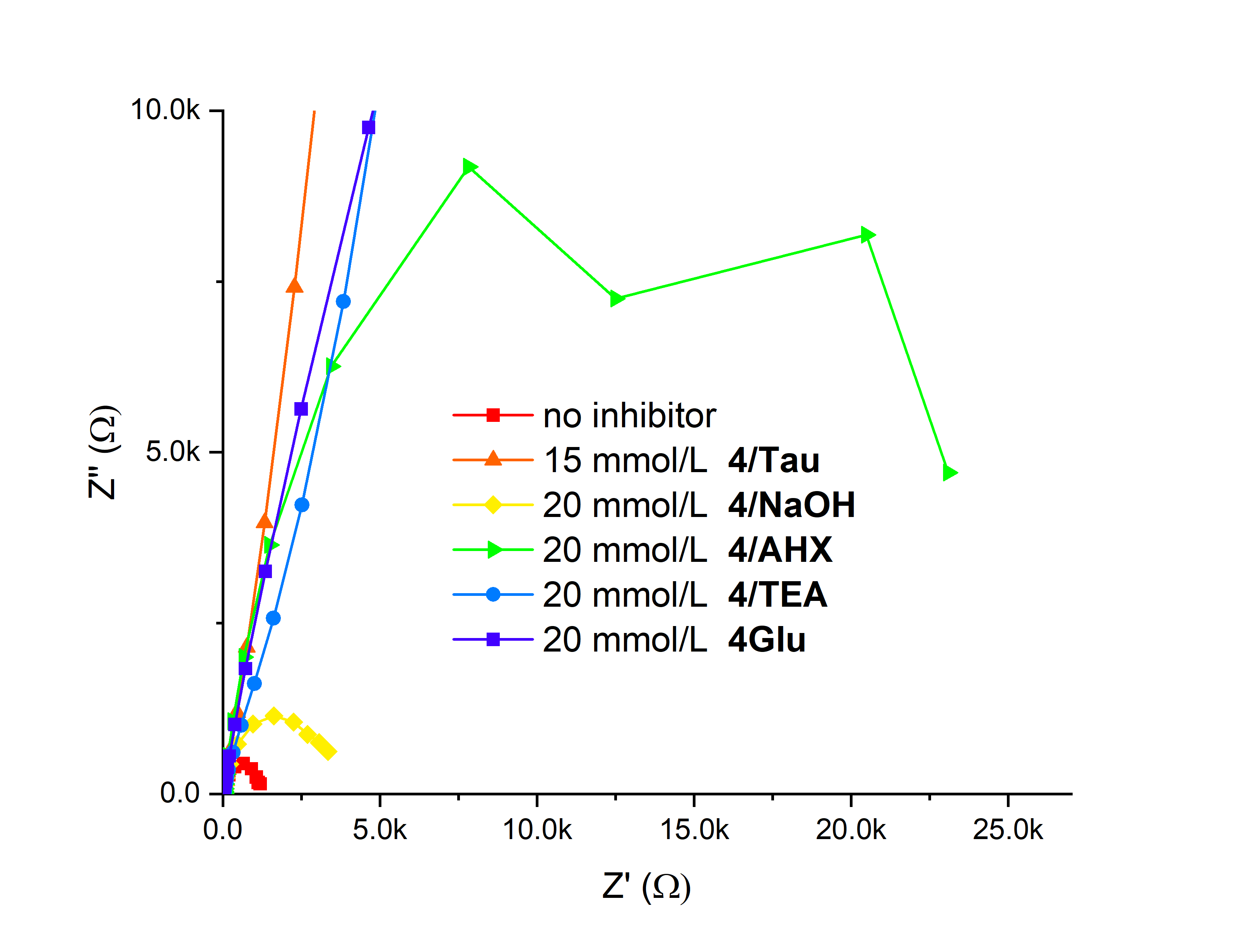 |  |
| NaCl-solution (0.5wt.%, pH 8.3 ± 0.5) |  |

## Fig. S5 Nyquist plots of electrochemical impedance measurements in detailed view

# **Example of DRT analysis**


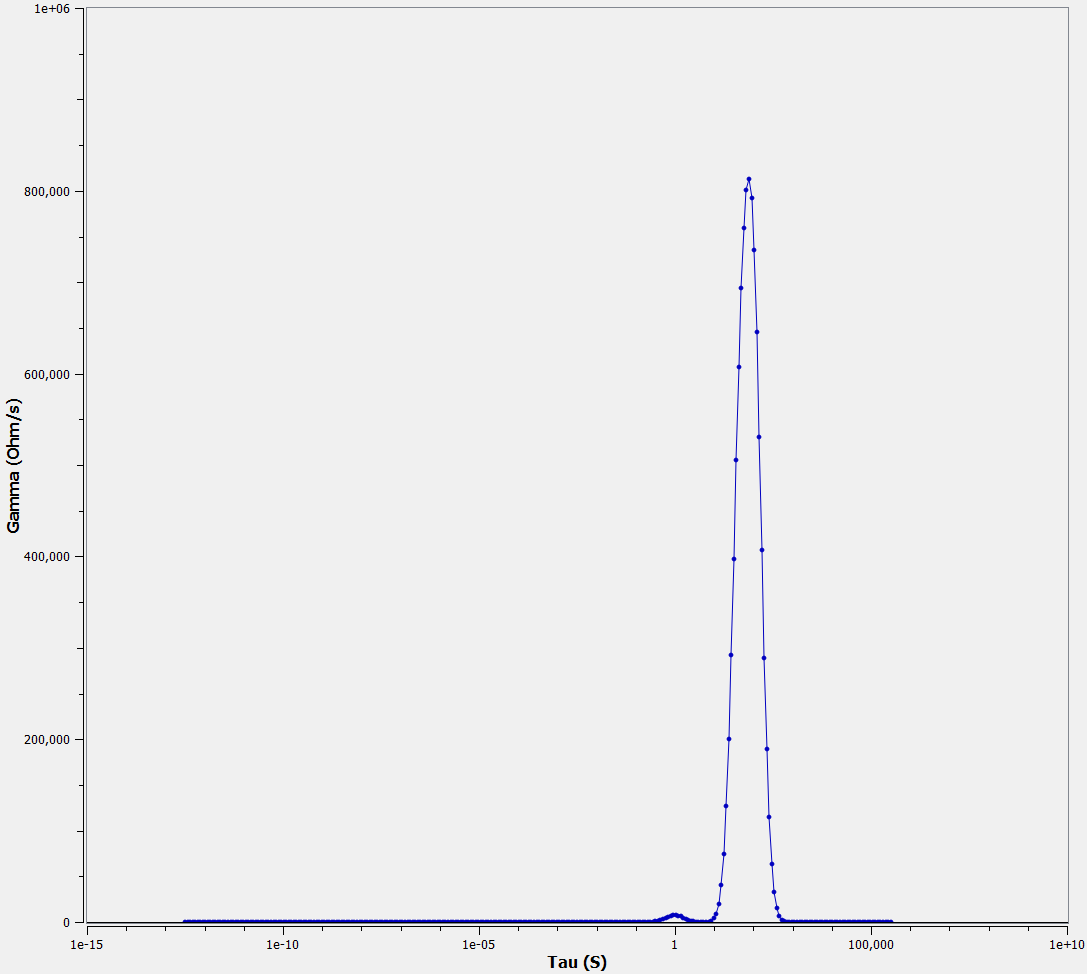


## Fig. S6 Exemplary results of the DRT analysis for 4 in 0.5 wt% NaCl solution after 24 h (table 3, entry 4). The analysis points to a system with mainly one R/C element. Further mechanistic investigations with an implementation of an equivalent circuit model will follow.

# **References**

Ruf E, Naundorf T, Seddig T, Kipphardt H, Maison W (2022) Natural Product-Derived Phosphonic Acids as Corrosion Inhibitors for Iron and Steel. Molecules 27(6):1778
